# Genome-Wide Characterization and Analysis of the bHLH Transcription Factor Family in *Suaeda aralocaspica*, an Annual Halophyte With Single-Cell C_4_ Anatomy

**DOI:** 10.3389/fgene.2022.927830

**Published:** 2022-07-07

**Authors:** Xiaowei Wei, Jing Cao, Haiyan Lan

**Affiliations:** Xinjiang Key Laboratory of Biological Resources and Genetic Engineering, College of Life Science and Technology, Xinjiang University, Urumqi, China

**Keywords:** genome-wide identification, bHLH, single-cell C_4_ anatomy, *Suaeda aralocaspica*, transcriptional expression

## Abstract

Basic helix-loop-helix (bHLH) transcription factors play important roles in plant growth, development, metabolism, hormone signaling pathways, and responses to abiotic stresses. However, comprehensive genomic and functional analyses of *bHLH* genes have not yet been reported in desert euhalophytes. *Suaeda aralocaspica*, an annual C_4_ halophyte without Kranz anatomy, presents high photosynthetic efficiency in harsh natural habitats and is an ideal plant for identifying transcription factors involved in stress resistance. In this study, 83 *bHLH* genes in *S. aralocaspica* were identified and categorized into 21 subfamilies based on conserved motifs, gene structures, and phylogenetic analysis. Functional annotation enrichment revealed that the majority of SabHLHs were enriched in Gene Ontology (GO) terms and Kyoto Encyclopedia of Genes and Genomes (KEGG) pathways involved in the response to stress conditions, as transcription factors. A number of *cis*-acting elements related to plant hormones and stress responses were also predicted in the promoter regions of *SabHLHs*, which were confirmed by expression analysis under various abiotic stress conditions (NaCl, mannitol, low temperature, ABA, GA_3_, MeJA, and SA); most were involved in tolerance to drought and salinity. SabHLH169 (076) protein localized in the nucleus was involved in transcriptional activity, and gene expression could be affected by different light qualities. This study is the first comprehensive analysis of the *bHLH* gene family in *S. aralocaspica.* These data will facilitate further characterization of their molecular functions in the adaptation of desert plants to abiotic stress.

## Introduction

Salinity, drought, and extreme temperatures are major abiotic stresses that severely affect plant growth and development. Plants have evolved adaptive strategies to cope with adverse environmental conditions, involving signaling cascades that activate transcription factors (TFs) (Mao et al., 2017). Various TFs act as molecular switches for abiotic stress tolerance by interacting with specific *cis*-elements in the promoters of stress-responsive genes (Yue et al., 2019). The basic helix-loop-helix (bHLH) gene family is one of the largest TF families in eukaryotes (Ledent and Vervoort, 2001) and is involved in plant growth, development, and stress tolerance (Groszmann et al., 2010; Deng et al., 2017; Guo J. et al., 2020). bHLH TFs are named for their highly conserved bHLH domain, which is composed of a basic region distributed at the N-terminus and a helix-loop-helix (HLH) structure located at the C-terminus. The basic region, containing approximately 17 amino acids, participates in recognition that allows bHLH TFs to bind to the conserved hexanucleotide E-box (5′-CANNTG-3′) or G-box (5′-CACGTG-3′) *cis*-element of the target gene promoter (Atchley et al., 1999; Toledo-Ortiz et al., 2003). The HLH region consists of 40–50 hydrophobic amino acids with two amphipathic α-helices separated by a divergent loop, and functions as a dimerization domain, promoting the formation of homodimers or heterodimers and interactions between proteins (Massari and Murre, 2000). In addition, some atypical bHLH TFs have been identified in *Arabidopsis thaliana*, which carry a few basic regions that are critical for DNA binding (Hyun and Lee, 2006; Roig-Villanova et al., 2007).

With the completion of genome sequencing for multiple species, numerous plant bHLH proteins have been characterized, including 162 proteins in *Arabidopsis* (Toledo-Ortiz et al., 2003), 167 in rice (Li et al., 2006), 208 in maize (Zhang et al., 2018), 437 in cotton (Lu et al., 2018), 571 in wheat (Wei and Chen, 2018), and 602 in *Brassica napus* (Ke et al., 2020). The functions of many plant bHLH proteins have been described in detail, indicating their involvement in regulating diverse physiological and biochemical processes, such as light signal transmission (Buti et al., 2020), plant hormone signaling (Seo et al., 2011), iron uptake (Ogo et al., 2007), anthocyanin and flavonoid biosynthesis (Xu et al., 2017; Tian et al., 2018), and stomatal, root, and petal growth (Ohashi-Ito and Bergmann, 2006; Szecsi et al., 2006; Kanaoka et al., 2008). For example, the *Arabidopsis* bHLH proteins PIF3 and PIF4 can directly interact with phytochrome in the signaling network of photoreceptors to control the expression of light-regulated genes (Toledo-Ortiz et al., 2003). Furthermore, PIF4 acts as a central hub, integrating multiple signals to regulate thermosensory growth and architectural adaptation in plants (Kumar et al., 2012). Notably, a substantial increase in the number of bHLH proteins has been demonstrated to play an important role in the response to abiotic stresses, including cold, drought, and salt stress. For example, in tomatoes, *SlPIF4* modulates cold tolerance in anthers via the temperature-dependent regulation of tapetal cell death (Pan et al., 2021). In rice, *OsbHLH148* regulates drought tolerance via jasmonic acid signaling pathway (Seo et al., 2011). In the halophyte *Chenopodium glaucum*, *CgbHLH001* could confer drought and salt tolerance to transgenic tobacco by improving its physiological performance in scavenging excess reactive oxygen species and accumulating the transcripts of stress-related genes (Zhou et al., 2020). Although diverse and important functions of bHLH TFs have been reported, limited information is available regarding bHLH TFs from C_4_ halophytes within desert communities. With the release of the draft genome assembly of *Suaeda aralocaspica*, it is now possible to identify genome-wide bHLH TFs in *S. aralocaspica* (Wang et al., 2019a).

*Suaeda aralocaspica* (Bunge) Freitag & Schütze (Amaranthaceae), an annual euhalophyte with succulent leaves, is restricted to saline-alkaline sandy soils in the inland cold desert of the Junggar Basin in China (Commissione Redactorum Florae Xinjiangensis 1994). To survive the harsh conditions of its natural habitat (e.g., high salinity, high light intensity, and high daily temperature variation), *S. aralocaspica* has evolved a series of adaptive strategies, including the production of three distinct seed morphs with differences in germination behavior and salt tolerance (Wang et al., 2008; Cao et al., 2021). In addition, it presents delayed development at the seedling stage to maintain the balance between development and energy consumption (Cao et al., 2022). Interestingly, *S. aralocaspica* was the first terrestrial plant species found to possess a unique C_4_ photosynthetic pathway without the Kranz anatomy, which enables photosynthesis to occur within an individual chlorenchyma cell (Voznesenskaya et al., 2001, 2004). These characteristics endow *S. aralocaspica* with abiotic tolerance and highly efficient photosynthesis (Liu et al., 2020); however, the molecular mechanism underlying stress tolerance remains poorly understood. Previously, we isolated the full-length (FL) cDNA sequence of the phosphoenolpyruvate carboxylase (PEPC), the key photosynthetic enzyme in *S. aralocaspica,* which was named *SaPEPC-1* (GenBank: KP985714.1), suggesting its role in development and stress tolerance in *S. aralocaspica* (Cheng et al., 2016; Cao et al., 2021). Recently, we identified a putative bHLH protein that may interact with the *SaPEPC-1* promoter from *S. aralocaspica,* termed SabHLH169 (homology to *Arabidopsis* bHLH169), using an *in vitro* DNA-pull-down method combined with liquid chromatography-mass spectrometry technology (unpublished data; Zheng et al., 2022). To date, the subfamily classification, transcriptional characteristics, and biological functions of this protein remain uncertain. Therefore, the major aims of the present study were to: 1) characterize all members of *bHLH* genes in *S. aralocaspica* at the genome-wide level; 2) compare functional differences among *SabHLH* isoforms in terms of phylogenetic relationships, gene structure, protein motifs, *cis*-acting elements, and protein interaction networks; and 3) investigate the contribution of different SabHLHs to development, hormones, and abiotic stress. This research should facilitate future studies on investigating the roles of bHLH isoforms in *S. aralocaspica* and other euhalophytes, which will improve our understanding of the molecular mechanisms of desert plant adaptation to environmental stress.

## Materials and Methods

### Sequence Retrieval and Identification of *bHLH* Genes in *S. aralocaspica*

The entire *S. aralocaspica* genome was obtained from the GigaScience GigaDB database (http://gigadb.org/dataset/100646 *;* Wang et al., 2019b). The Hidden Markov Model (HMM)-based profile of the bHLH domain (PF00010) downloaded from the Pfam database (http://pfam.xfam.org/) was used as a query to scan the *S. aralocaspica* proteome file using HMMER (http://hmmer.janelia.org/) with a default *E*-value. The online CD-search tool (https://www.ncbi.nlm.nih.gov/Structure/bwrpsb/bwrpsb.cgi) and SMART database (http://smart.embl-heidelberg.de/) were used to verify the existence of the conserved bHLH domain in the putative *S. aralocaspica* bHLH proteins. Redundant sequences were removed manually, and the identified *bHLH* genes were named according to their order in the *S. aralocaspica* genomic sequence. The length, theoretical molecular weight (MW), and isoelectric point (*pI*) of SabHLH candidates were predicted using ExPASy (http://web.expasy.org/compute_pi/).

### Phylogenetic Analysis of bHLH Proteins in *S. aralocaspica*

Multiple alignments were performed using FL amino acid sequences of putative bHLH proteins in *S. aralocaspica* with the ClustalW program of MEGA X using default settings (Kumar et al., 2018). A phylogenetic tree of bHLH proteins from *S. aralocaspica* and *Arabidopsis* was constructed using the unrooted Neighbor-Joining (NJ) method of MEGA X with the following parameters: Poisson correction, pairwise deletion, and a bootstrap analysis with 1,000 replicates. The resultant Newick tree output file was visualized using iTOL v.5 (https://itol.embl.de/). Supplementary Table S1 provides a detailed description of previously used *Arabidopsis* proteins and their corresponding accession numbers.

### Analysis of Gene Structures, Conserved Motifs, and *Cis*-Regulatory Elements

To analyze gene structure, the exons and introns of *SabHLH* genes were identified from the alignment of cDNA sequences with the corresponding genomic DNA sequences and illustrated using the TBtools software (Chen et al., 2020). MEME Version 5.4.1 software (http://meme-suite.org/tools/meme) was employed to identify and analyze the conserved motifs of candidate SabHLH proteins with the following parameters: number of motifs to find, 10; minimum width of motifs, 6; and maximum width of motifs, 250. The PlantCARE database (http://bioinformatics.psb.ugent.be/webtools/plantcare/html/) was used to analyze the *cis*-regulatory elements in the promoter sequences (2,000 bp upstream of the start codon) of *SabHLH* genes. The results from these analyses of gene structure, conserved motifs, and *cis*-acting elements were arranged according to the order shown in the phylogenetic tree using TBtools software (Chen et al., 2020).

### Protein Association Network Predictions and Functional Classification

All putative SabHLH protein sequences were submitted to the STRING online server (http://string-db.org) to construct a network of functionally interacting orthologous genes between *S. aralocaspica* and *Arabidopsis,* with default parameters. Genes that did not interact with any other gene were excluded. Gene Ontology (GO) and Kyoto Encyclopedia of Genes and Genomes (KEGG) pathway annotations were performed by submitting all SabHLH protein sequences to the eggNOG-mapper online website (http://eggnog-mapper.embl.de/) and visualized using TBtools software (Chen et al., 2020).

### Expression Profile Analysis of *bHLH* Genes in *S. aralocaspica*

The expression profiles of *bHLH* genes in *S. aralocaspica* were analyzed based on publicly released data. RNA-seq datasets for different tissues (BioProject: JNA428881; Wang et al., 2019a) and for dimorphic seeds during germination (BioProject: PRJNA325861; Wang L. et al., 2017) were downloaded from the NCBI Sequence Read Archive (SRP128359). Gene expression levels were estimated as fragments per kilobase of exons per million mapped reads (FPKM) using Cufflinks software (Trapnell et al., 2012). A heatmap was generated using TBtools software (Chen et al., 2020); the color scale represents FPKM counts, and the ratios were log_2_ transformed.

### Plant Materials and Treatments

Mature *S. aralocaspica* seeds were harvested from dry inflorescences in natural populations growing in the Gurbantunggut Desert at Wujiaqu 103 regiment (44°29′N, 87°31′E; 430 mH) in October 2017 in the Xinjiang Uygur Autonomous Region, China. The seeds were air-dried indoors, cleaned, and stored at 4°C in sealed brown paper bags. To collect samples for total RNA extraction, approximately 150 brown seeds were sown on two layers of moist filter paper in a 15 cm Petri dish and exposed to different treatment conditions. For different germination times, germinating seeds (seedlings) were harvested at 8, 12, 24 h, 2, 5, 10, and 15 days, and dry seeds at 0 h were used as the control. For different tissues, cotyledons, hypocotyls and radicles were harvested from 7-day-old seedlings, and leaves, stems, and roots were collected from 30-day-old mature plants. For salinity and drought stress, filter paper was saturated with 20 ml of distilled water (used as a control) or different concentrations of aqueous solutions, NaCl (100, 300, and 500 mmol L^−1^) and PEG6000 [5%, 10% (v/v)], and seedlings were harvested after germination for 7 days. For different light qualities, the Petri dishes were covered with red, yellow, green, blue, and transparent (white light) plastic filters, respectively (Montero et al., 2016), and wrapped with tinfoil to prevent light penetration (darkness). All Petri dishes were placed in a 24-h normal light incubator, and the seedlings were harvested after 7-day germination. To simulate different hormone stress conditions, filter paper was saturated with 20 ml of the following aqueous solutions: ABA (0.5 μmol L^−1^), GA_3_ (800 mg mL^−1^), SA (1.5 mg mL^−1^), or MeJA (0.5 μmol L^−1^); seedlings were harvested at 8 h and 3 days, and dry seeds harvested at 0 h were used as the control. All Petri dishes were maintained in an illumination incubator (RXZ-5000C, Ningbo Jiangnan Instrument Factory, China) at a constant temperature of 25°C under a photoperiod of 16 h light/8 h dark, with a light intensity of 500 μmol m^−2^·s^−1^. For low-temperature stress, all Petri dishes were placed in a 4°C illumination incubator, in which light intensity and photoperiod remained unchanged, and the seedlings were harvested at 0 (dry seeds), 2, 4, 8, and 12 h, respectively. All samples were immediately frozen in liquid nitrogen for harvesting and then stored at -80°C until use. Three biological replicates were used per treatment.

### Quantitative Real-Time PCR Analysis

Total RNA was extracted from seedlings using the E.Z.N.A^®^ Plant RNA Kit (Cat. R6827, OMEGA, United States), according to the manufacturer’s instructions. Each reverse transcription reaction was performed with 1 µg of total RNA in a final volume of 20 µl using the M-MLV RTase cDNA Synthesis Kit (D6130, TaKaRa, Shiga, Japan) with 2.5 μmol L^−1^ oligo (dT) primer, according to the manufacturer’s instructions. The cDNA was stored at -20°C until use. qRT-PCR was performed using BlasTaq^TM^ 2X qPCR MasterMix (abm, Zhenjiang, China) in the LightCycler 96 Real-Time System (Roche, Basel, Switzerland). The relative expression of the *β-tubulin* gene of *S. aralocaspica* was used for normalization (Cao et al., 2016). The primers used for qRT-PCR analysis are listed in Supplementary Table S2. The reaction mixture consisted of 1 μl of cDNA, 0.5 μl each of forward and reverse primers, 10 μl of BlasTaq^TM^ qPCR master mix, and 8 μl of RNase-free water in a final volume of 20 µl. The reaction conditions for qRT-PCR were as follows: initial denaturation at 94°C for 30 s, followed by 40 cycles of denaturation at 94°C for 5 s, and annealing at 60°C for 30 s. The relative expression levels of candidate genes were determined according to the mathematical model: *R* = 2^−∆∆CT^ (Shi and Chiang, 2005), where ∆∆CT = ∆CT_target_ _sample_ − ∆CT_control_ _sample_, and ∆CT_sample_ = CT_test gene_ − CT_reference gene_. Relative quantification was presented as the normalized fold-change in gene expression of each target gene compared to the control. Data are expressed as the mean ± standard error of three biological replicates and two technical replicates (*n* = 6).

### Determination of Subcellular Localization

The Plant-mPLoc website (http://www.csbio.sjtu.edu.cn/bioinf/plant-multi/) was used to predict the subcellular localization of total candidate bHLHs in *S. aralocaspica*. This was verified by transient expression of the SabHLH169 (identified as SabHLH076 in the present study) fusion protein in tobacco epidermal cells. The open reading frame sequence of *SabHLH169(076)* (with the stop codon deletion) was cloned into the pSuper1300-*MCS*-*eGFP* plant expression vector, which was then transformed into *Agrobacterium tumefaciens* strain GV3101 using the CaCl_2_ method (Holsters et al., 1978). Primers used for vector construction are listed in Supplementary Table S2. Correct single colonies were cultivated, harvested, and resuspended in infiltration buffer (10 mmol L^−1^ MES, 10 mmol L^−1^ MgCl_2_ and 150 μmol L^−1^ acetosyringone) at a final concentration of OD_600_ = 0.8. The *A. tumefaciens* suspension (A) of the abovementioned construct was evenly mixed with pSuper1300-*P19*/GV3101 suspensions (B) (P19 protein: promoted protein expression) in a volume ratio of 500 μl (A): 500 μl (B) and then held at room temperature for 2 h in the dark before use. Approximately 5–6-week-old *Nicotiana benthamiana* plants were used for infiltration. A mixture of different *Agrobacterium* strains was infiltrated into fresh leaves, and the infiltration areas were labelled for recognition (Wang J. et al., 2017). Treated plants were left in the dark overnight and then transferred to normal growth conditions for 72 h. Cell nuclei were visualized with DAPI, which was injected into the marked area with a syringe after which plants were incubated in the dark for 4 h. The fluorescent signals in *N. benthamiana* leaves were visualized using a Zeiss LSM 800 confocal microscope (Carl Zeiss, Jena, Germany).

### Transactivation Assay Analysis

The cDNA sequence of *SabHLH169(076)* was inserted into the pGBKT7 yeast expression vector. The pGBKT7-*SabHLH169(076)*, pGBKT7 (negative control), and pGBKT7-*CgbHLH001* (positive control; Zhou et al., 2020) plasmids were transformed into competent AH109 yeast cells. The transformed yeast cells were spread on yeast synthetic dropout medium without Trp and His (SD/-His-Trp) and incubated at 30°C for 3 days. To confirm activation of the reporter gene *alpha-galactosidase*, the X-alpha-gal colony-lift filter assay was performed according to the protocols provided in the Yeast Protocol Handbook (Clontech, Mountain View, CA, United States).

### Statistical Analysis

All data were plotted using GraphPad Prism version 7.0 (GraphPad Software, San Diego, CA, United States) and analyzed using SPSS version 26.0 (SPSS Inc., Chicago, IL, United States). Univariate scatterplots displaying parametric data were presented as the mean ± standard deviation (SD) (Weissgerber et al., 2015). One-way ANOVA was performed to test the significance of different treatments, and Tukey’s HSD test was performed for multiple comparisons to determine significant differences between samples at 0.05 significance level. When the homogeneity of variance assumption was not met, differences were analyzed using Welch’s ANOVA and Games-Howell post-hoc tests (McDonald, 2014).

## Results

### Identification and Characterization of *bHLH* Gene Family in *S. aralocaspica*

For genome-wide identification of *SabHLH* genes, the HMM file was used as a query to search for the *S. aralocaspica* proteome. All candidate sequences were filtered using SMART and CD-Search to confirm that they contained a complete bHLH domain. Overall, 83 *SabHLHs* were identified and named *SabHLH001* to *SabHLH083* in accordance with their order in the *S. aralocaspica* genomic sequence (Wang et al., 2019b). The SabHLH family members varied markedly in terms of protein sequence length, from 91 (SabHLH024) to 699 [SabHLH169 (076)] amino acids (aa), with an average length of 370 aa. The molecular weights of the proteins ranged from 10.031 kDa (SabHLH048) to 77.395 kDa [SabHLH169 (076)], and their theoretical *pI* values ranged from 4.47 (SabHLH048) to 10.91 (SabHLH061). Predictions of subcellular localizations revealed that all SabHLH proteins were localized only in the nucleus, except for SabHLH038 was located in the chloroplast and nucleus, and SabHLH044 was located in the cytoplasm. Detailed information on *SabHLH* family members is shown in Table 1.

**TABLE 1:** Characteristics of *bHLH* gene family in *S. aralocaspica*.

Gene name	Genome ID	GenBank	ORF (bp)	*pI*	MW (kDa)	Size (aa)	Subcellular location
SabHLH001	GOSA_00000076-RA	ON400862	1,668	5.74	60.27532	556	Nucleus
SabHLH002	GOSA_00000223-RA	ON400863	1,383	6.67	50.40351	461	Nucleus
SabHLH003	GOSA_00000435-RA	ON400865	1,431	4.72	53.86281	477	Nucleus
SabHLH004	GOSA_00000601-RA	ON400861	2,037	6.16	74.15591	679	Nucleus
SabHLH005	GOSA_00000643-RA	ON400860	1,431	6.28	52.52881	477	Nucleus
SabHLH006	GOSA_00000671-RA	ON400866	1,020	8.66	38.82797	340	Nucleus
SabHLH007	GOSA_00000723-RA	ON400867	885	6.13	31.33137	295	Nucleus
SabHLH008	GOSA_00001077-RA	ON400864	1,122	5.71	41.83251	374	Nucleus
SabHLH009	GOSA_00001306-RA	ON400858	891	7.75	33.91263	297	Nucleus
SabHLH010	GOSA_00001307-RA	ON400856	852	5.55	31.92971	284	Nucleus
SabHLH011	GOSA_00001444-RA	ON400857	648	6.36	24.71007	216	Nucleus
SabHLH012	GOSA_00001689-RA	ON400859	1,992	5.91	72.36971	664	Nucleus
SabHLH013	GOSA_00001701-RA	ON400855	906	8.6	34.04228	302	Nucleus
SabHLH014	GOSA_00001967-RA	ON400888	1,494	5.24	53.81603	498	Nucleus
SabHLH015	GOSA_00001974-RA	ON400884	576	6.85	21.2707	192	Nucleus
SabHLH016	GOSA_00002069-RA	ON400887	1,188	5.98	45.26038	396	Nucleus
SabHLH017	GOSA_00002373-RA	ON400883	1,125	5.62	41.93782	375	Nucleus
SabHLH018	GOSA_00002416-RA	ON400885	1,362	7.1	51.12012	454	Nucleus
SabHLH019	GOSA_00002694-RA	ON400886	1,341	6.19	47.16991	447	Nucleus
SabHLH020	GOSA_00002849-RA	ON400893	735	5.39	27.55012	245	Nucleus
SabHLH021	GOSA_00003472-RA	ON400892	624	9.17	23.1888	208	Nucleus
SabHLH022	GOSA_00003586-RA	ON400872	843	8.29	31.46418	281	Nucleus
SabHLH023	GOSA_00003869-RA	ON400871	1,053	8.34	38.86735	351	Nucleus
SabHLH024	GOSA_00004355-RA	ON400904	273	7.94	10.50083	91	Nucleus
SabHLH025	GOSA_00004508-RA	ON400876	1,005	4.9	37.11856	335	Nucleus
SabHLH026	GOSA_00004680-RA	ON400877	1,515	7.09	55.49633	505	Nucleus
SabHLH027	GOSA_00004781-RA	ON400878	1,983	4.64	75.61593	661	Nucleus
SabHLH028	GOSA_00004882-RA	ON400879	1,263	8.93	44.33566	421	Nucleus
SabHLH029	GOSA_00005410-RA	ON400896	1,206	6.05	44.62922	402	Nucleus
SabHLH030	GOSA_00005475-RA	ON400897	945	8.79	35.53922	315	Nucleus
SabHLH031	GOSA_00006132-RA	ON400869	927	6.16	33.86087	309	Nucleus
SabHLH032	GOSA_00006633-RA	ON400870	777	5.49	28.83567	259	Nucleus
SabHLH033	GOSA_00006634-RA	ON400868	822	5.26	30.63455	274	Nucleus
SabHLH034	GOSA_00006830-RA	ON400913	1,257	5.76	46.51908	419	Nucleus
SabHLH035	GOSA_00007033-RA	ON400912	726	7.58	26.98551	242	Nucleus
SabHLH036	GOSA_00007093-RA	ON400850	1,131	6.27	41.48272	377	Nucleus
SabHLH037	GOSA_00007353-RA	ON400851	2,067	6.45	74.6124	689	Nucleus
SabHLH038	GOSA_00008204-RA	ON400849	927	6.4	32.26992	309	Chloroplast\Nucleus
SabHLH039	GOSA_00008334-RA	ON400848	1,254	5.14	47.27305	418	Nucleus
SabHLH040	GOSA_00008687-RA	ON400874	963	5.96	35.63039	321	Nucleus
SabHLH041	GOSA_00008720-RA	ON400875	1,011	6.26	36.6356	337	Nucleus
SabHLH042	GOSA_00010211-RA	ON400903	1,206	6.1	44.07468	402	Nucleus
SabHLH043	GOSA_00010462-RA	ON400902	780	9.38	28.2595	260	Nucleus
SabHLH044	GOSA_00010702-RA	ON400889	1,980	5.31	72.66926	660	Cytoplasm
SabHLH045	GOSA_00010732-RA	ON400891	1,206	6.54	45.62066	402	Nucleus
SabHLH046	GOSA_00010765-RA	ON400890	987	4.76	37.56619	329	Nucleus
SabHLH047	GOSA_00011122-RA	ON400919	1,209	5.56	42.58493	403	Nucleus
SabHLH048	GOSA_00011171-RA	ON400918	258	4.47	10.03095	86	Nucleus
SabHLH049	GOSA_00011177-RA	ON400916	762	5.4	29.04995	254	Nucleus
SabHLH050	GOSA_00011282-RA	ON400917	690	6.6	25.60963	230	Nucleus
SabHLH051	GOSA_00011283-RA	ON400920	960	6.13	35.77709	320	Nucleus
SabHLH052	GOSA_00011847-RA	ON400911	1,194	8.63	43.00472	389	Nucleus
SabHLH053	GOSA_00011938-RA	ON400910	1,389	9.43	50.93376	463	Nucleus
SabHLH054	GOSA_00012457-RA	ON400926	960	5.36	35.7185	320	Nucleus
SabHLH055	GOSA_00012983-RA	ON400923	792	5.48	30.25622	264	Nucleus
SabHLH056	GOSA_00013009-RA	ON400922	390	5.06	14.57895	130	Nucleus
SabHLH057	GOSA_00013011-RA	ON400921	804	8.37	30.67592	268	Nucleus
SabHLH058	GOSA_00013158-RA	ON400908	1,017	5.35	37.85607	339	Nucleus
SabHLH059	GOSA_00013161-RA	ON400907	963	4.95	36.1451	321	Nucleus
SabHLH060	GOSA_00013229-RA	ON400906	1,203	6.24	43.18755	401	Nucleus
SabHLH061	GOSA_00013409-RA	ON400894	471	10.91	17.91746	157	Nucleus
SabHLH062	GOSA_00014741-RA	ON400873	1,584	9.3	59.38059	528	Nucleus
SabHLH063	GOSA_00015033-RA	ON400852	933	5.93	33.2459	311	Nucleus
SabHLH064	GOSA_00015228-RA	ON400914	1,479	5.57	55.14247	493	Nucleus
SabHLH065	GOSA_00015812-RA	ON400905	441	10.26	16.50293	147	Nucleus
SabHLH066	GOSA_00016476-RA	ON400909	813	7.63	31.04324	271	Nucleus
SabHLH067	GOSA_00018427-RA	ON400853	1,008	5.68	37.65921	336	Nucleus
SabHLH068	GOSA_00018432-RA	ON400854	1,068	6.95	39.4324	356	Nucleus
SabHLH069	GOSA_00018520-RA	ON400887	828	9.14	30.15356	276	Nucleus
SabHLH070	GOSA_00018672-RA	ON400927	1,023	6.27	37.64574	341	Nucleus
SabHLH071	GOSA_00018673-RA	ON400928	1,188	5.84	33.1677	296	Nucleus
SabHLH072	GOSA_00018924-RA	ON400929	1,017	5.73	37.30524	339	Nucleus
SabHLH073	GOSA_00019477-RA	ON400901	765	5.77	27.0998	255	Nucleus
SabHLH074	GOSA_00019526-RA	ON400898	1,503	5.97	55.38031	501	Nucleus
SabHLH075	GOSA_00019527-RA	ON400900	1,530	5.97	56.18658	510	Nucleus
SabHLH076	GOSA_00019547-RA	ON400899	2,097	5.96	77.3953	699	Nucleus
SabHLH077	GOSA_00020011-RA	ON400924	1,071	4.83	38.69557	357	Nucleus
SabHLH078	GOSA_00020455-RA	ON400895	1,929	5.43	73.52925	643	Nucleus
SabHLH079	GOSA_00020680-RA	ON400925	1,791	5.3	65.85017	597	Nucleus
SabHLH080	GOSA_00022456-RA	ON400882	1,416	5.47	52.09624	472	Nucleus
SabHLH081	GOSA_00022501-RA	ON400880	1,593	5.62	57.19449	531	Nucleus
SabHLH082	GOSA_00022531-RA	ON400881	747	6.61	28.33789	249	Nucleus
SabHLH083	GOSA_00026973-RA	ON400915	945	7	35.06321	315	Nucleus

### Phylogenetic Analysis of bHLHs in *S. aralocaspica*

**FIGURE 1:**
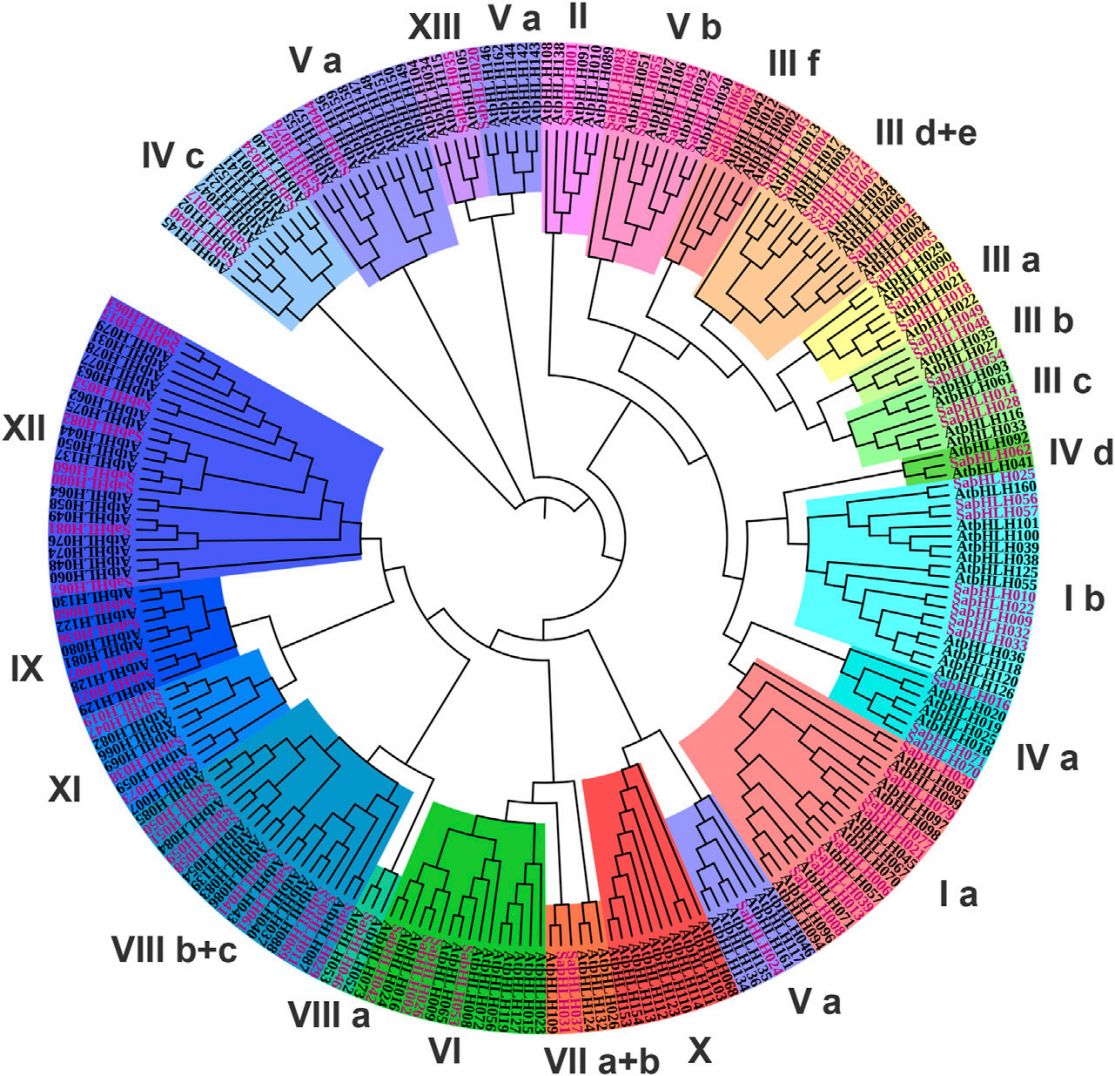
Phylogenetic tree and subfamily classifications of SabHLH and AtbHLH proteins. The conversed protein sequences of a total of 83 SabHLHs and 162 AtbHLHs were used to construct the phylogenetic tree using the Neighbor-Joining method in MEGA X with 1,000 bootstrap replications. The different subfamilies numbered from I to XIII are marked using different color backgrounds. All SabHLH proteins are shown in purple font.

To investigate the evolutionary relationships among SabHLH proteins, an unrooted NJ phylogenetic tree was constructed using the FL amino acid sequences of 83 SabHLH and 162 AtbHLH proteins (Supplementary Table S1). The SabHLH members were clustered into 21 subfamilies based on tree topology and classification of the bHLH superfamily in *Arabidopsis*, which is consistent with the finding that the bHLH family can be divided into 15–25 subfamilies (Toledo-Ortiz et al., 2003). The 21 subfamilies were designated as I a, I b, II, III a, III b, III c, III d+e, III f, IV a, IV c, IV d, V a, V b, VI, VII a + b, VIII a, VIII b + c, IX, XI, XII, and XIII (Figure 1). Subfamily VIII b+c contained the largest number of SabHLHs (9) and AtbHLHs (11), while subfamilies XIII, II, IV d, and VIII a were smaller, containing only one SabHLH, with the remaining subfamily containing 2–8 SabHLH members. No SabHLHs were found in subfamily X, possibly due to the loss of individual *SabHLH* genes during evolution.

### Gene Structure and Conserved Motif Analysis of *SabHLHs*

**FIGURE 2:**
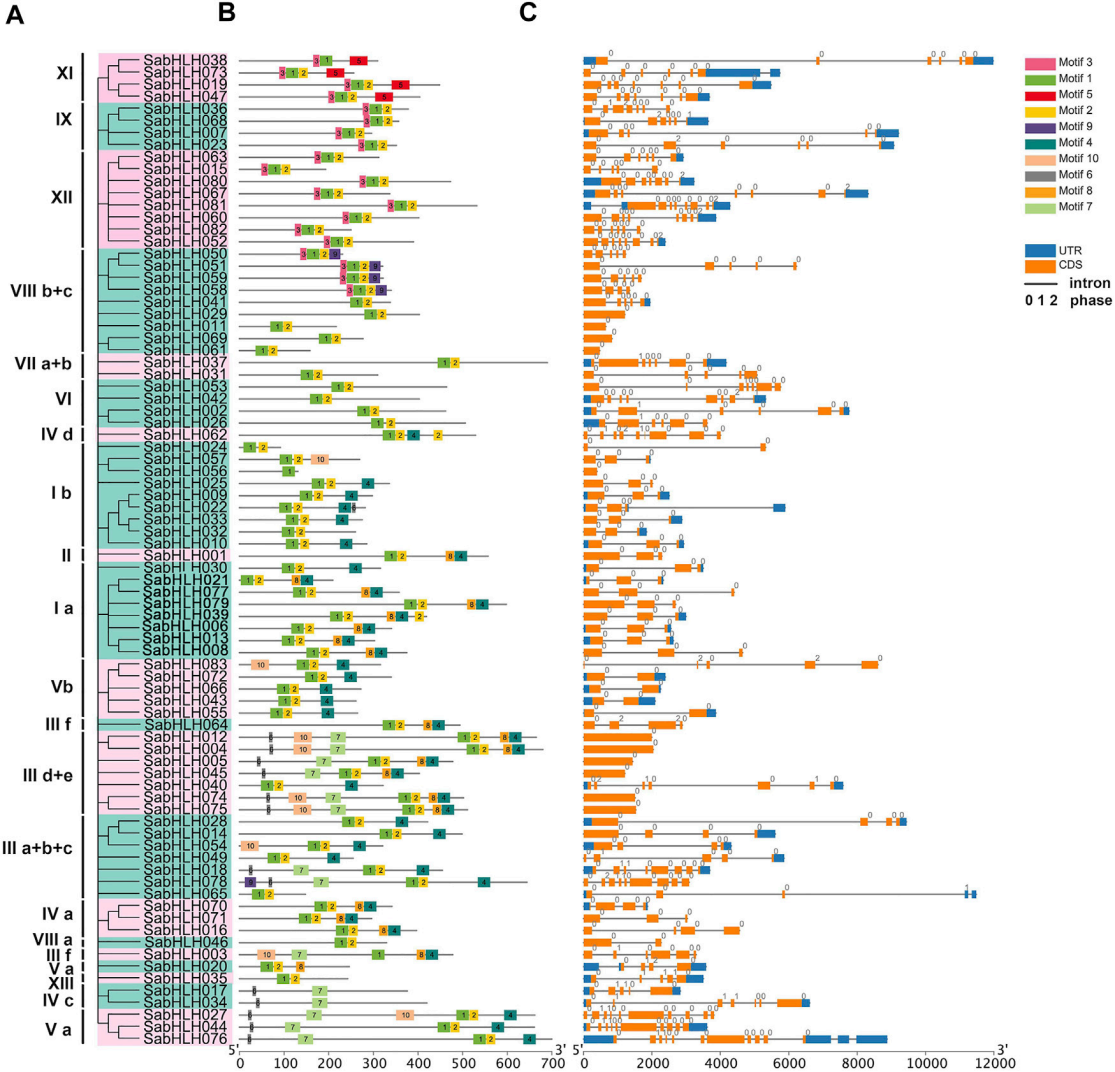
Phylogenetic relationships, motifs analysis, and gene structure of *bHLHs* in *S. aralocaspica*. **(A)** Rootless Neighbor-Joining (NJ) phylogenetic tree of 83 full-length amino acid sequences of SabHLH proteins. The different subfamilies numbered from I to XIII were marked using different color backgrounds. **(B)** Conserved motifs of SabHLH proteins. Different motifs are indicated by different color boxes numbered 1–10, and motif sizes can be estimated by the scale (bp) at the bottom. **(C)** Exon-intron structure of *SabHLH* genes. Grey lines represent introns, orange boxes represent exons, and blue boxes represent untranslated regions (UTRs). The numbers 0, 1, and 2 represent the intron phases.

Structure analyses revealed that different isoforms of *SabHLH* genes exhibited large differences in their exon/intron structures, and the number of introns varied from 0 to 12, corresponding to one to 11 exons (Figure 2). While most *SabHLH* genes contained one to eight introns, 34 *SabHLH* genes were intron-less and distributed across all subfamilies, particularly subfamilies VIII b+c and IIId+e, which contain four intron-less genes (Figure 2C). Ten conserved motifs were identified in the *SabHLH* gene family, and the length of the conserved motifs ranged from nine (motif 7) to 41 (motifs 5 and 10) amino acids (Figure 2B). These patterns of motif composition tended to be consistent with the phylogenetic tree, and the *SabHLHs* within each subfamily shared similar motif compositions; however, these varied greatly among different subfamilies. Most sequences exhibited two types of highly conserved protein motifs: green (motif 1) and yellow (motif 2) blocks, representing the position of the bHLH domain. Some of the other eight motifs were present only in certain groups, such as motif 5 present in superfamily XI, motif 9 in superfamily VIII b + c, motif 10 in superfamilies I b, V b, III d + e, III a + b + c, and III f; motif 6 in superfamilies III d + e, IV c, and V a; and motif 3 in superfamilies IX and XII. Superfamily III d + e possessed the most motifs, and genes in the other groups presented more complex structures. Different motifs may be related to the unique functions of the individual subfamilies.

### *Cis*-Acting Regulatory Element Analysis of *SabHLH* Promoters

The retrieved 2,000 bp sequences upstream of the start codon of *SabHLH* genes were queried to predict *cis*-regulatory elements using the PlantCARE database. Fourteen varieties of *cis*-elements were detected from 83 *SabHLHs* genes, which are involved in the response to light (i.e., G-box, GT1-motif, MRE, and ACE), phytohormones [ABA (abscisic acid, ABRE), JA (methyl jasmonate, CGTCA-motif), GA_3_ (gibberellin, GARE-motif), SA (salicylic acid, TCA-element), and auxin (TGA-element)], abiotic stresses [including drought (MBS), low temperature (LTR), and wound-responsive (WUN-motif)], defense- and stress-responsive (TC-rich repeats), anoxic specific inducibility (GC-motif), biotic stress (anaerobic induction, ARE), circadian control (circadian, CAAAGATATC), and seed-specific regulation (RY-element) (Supplementary Figure S1). The number of *cis*-acting elements involved in the light response was the highest, followed by hormone and stress responses. Notably, two genes, *SabHLH056* and *SabHLH057*, contained the largest numbers of low temperature-response and JA-response elements, respectively. These findings indicated that the *SabHLHs* family may participate in various plant hormone signaling pathways and are linked to stress resistance, plant growth, and development in *S. aralocaspica*.

### Interaction Network Prediction and Functional Classification of SabHLHs

**FIGURE 3:**
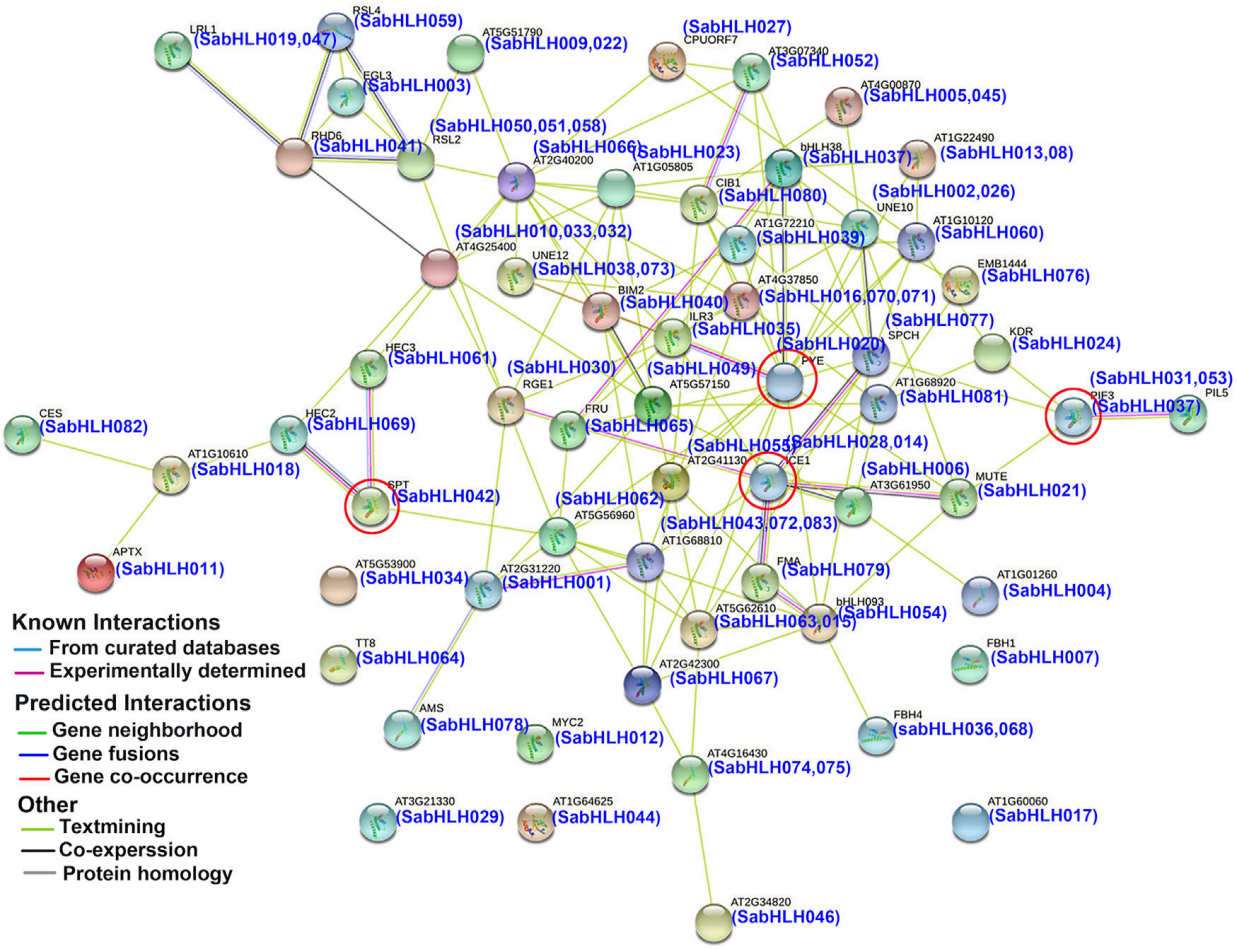
Protein interaction network for SabHLHs in *S. aralocaspica* according to orthologs in *Arabidopsis*. The online tool STRING was used to predict the network. SabHLH proteins are shown in brackets with *Arabidopsis* orthologs. Line and node colors indicate the different types and degrees of interactions, respectively. The filled or empty nodes represent known or unknown 3D structures, respectively. The red circle represents several important interactions.

To predict the functions of SabHLHs, a putative interaction network was constructed using the STRING database based on ortholog proteins in *Arabidopsis* (Figure 3), which is consistent with previous reports that the binding activity of bHLH proteins depends on the formation of homodimers or heterodimers among bHLH proteins (Carretero-Paulet et al., 2010). A total of 58 SabHLH proteins had orthologs in *Arabidopsis*, and 52 SabHLHs were predicted to have a protein-interaction relationship. Overall, several important interactions were predicted, as shown in Figure 3. ICE1 (homolog of SabHLH014 and SabHLH028) can interact with FMA (homolog of SabHLH079), SPCH (homolog of SabHLH077), and MUTE (homolog of SabHLH021) to regulate stomatal differentiation in *Arabidopsis* (Marcos et al., 2017). PIF3 (homolog of SabHLH037) interacts with PIL5 (homolog of SabHLH031 and SabHLH051), which is involved in the regulation of plant photomorphogenesis (Bu et al., 2011). PYE (homolog of SabHLH020) interacts with ILR3 (homolog of SabHLH035) to maintain iron homeostasis under low-iron conditions and positively regulates growth and development under iron-deficient conditions (Long et al., 2010; Selote et al., 2015). HEC2 (homolog of SabHLH069) and HEC3 (homolog of SabHLH061) can interact with SPT (homolog of SabHLH042) to regulate pistil development (Gremski et al., 2007). These results illustrate the functional diversity of TF genes. Although further experiments are needed to evaluate all potential interactions, the predicted network provides insights for studies on the functions of SabHLH family members.

**FIGURE 4:**
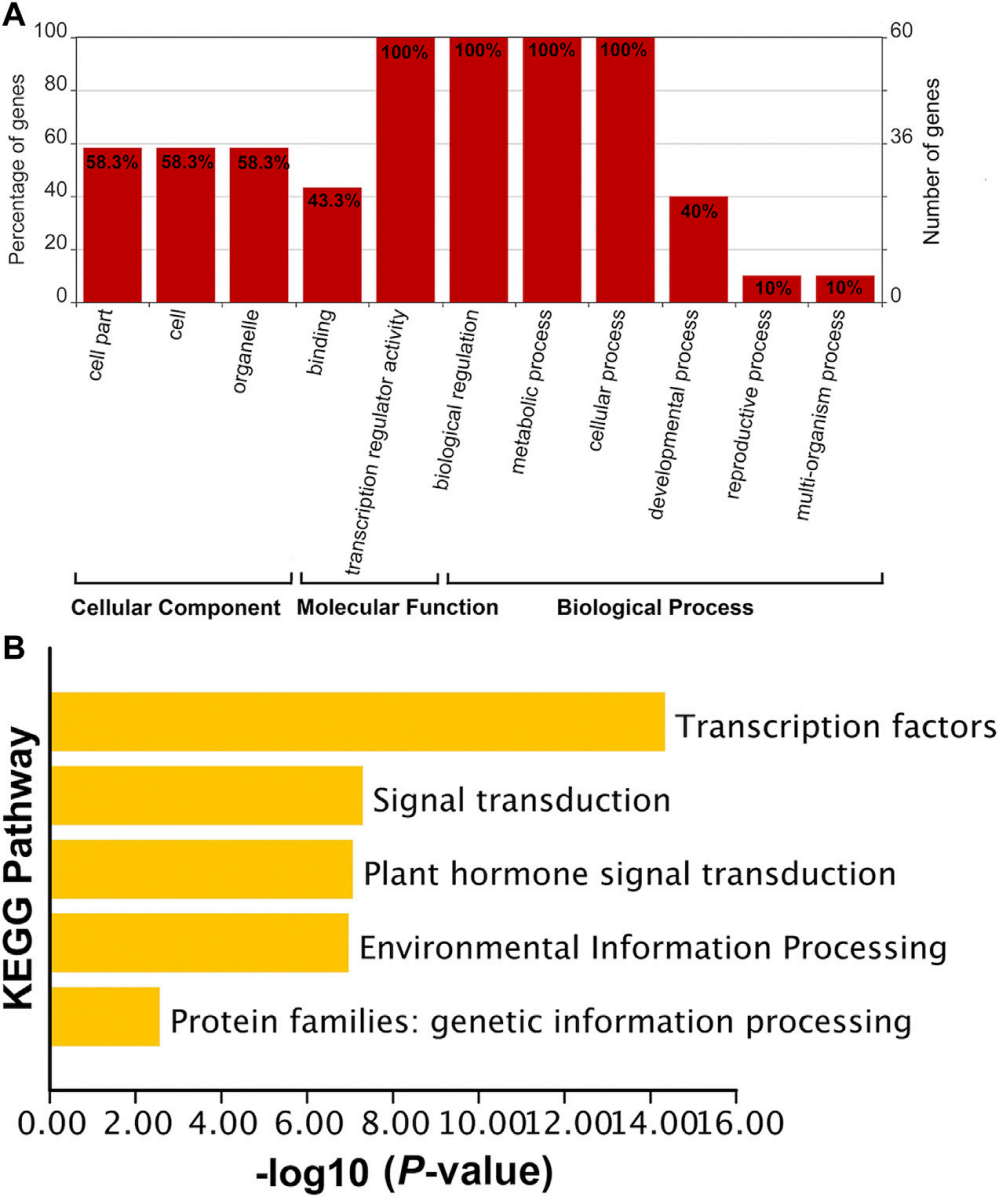
Functional annotation of *SabHLH* genes. **(A)** Gene Ontology (GO) annotation of 83 *SabHLH* proteins according to the three categories biological process (BP), cellular component (CC), and molecular function (MF). **(B)** Kyoto Encyclopedia of Genes and Genomes (KEGG) pathway annotation of *SabHLH* genes in *S. aralocaspica*.

The functions of *SabHLH* genes were subjected to GO and KEGG analyses (Figure 4). GO functions are divided into three aspects: cell component (CC), molecular function (MF), and biological process (BP). The MF and BP aspects mainly describe the molecular activities of multiple genes, and the CC aspect describes the locations where gene products are active. In the MF category, *SabHLH* genes were significantly enriched for binding (*n* = 26, 43.3%) and transcription regulator activity (*n* = 60, 100%). In the BP category, genes were enriched in biological regulation (*n* = 60, 100%), metabolic processes (*n* = 60, 100%), cellular processes (*n* = 60, 100%), developmental processes (*n* = 24, 40%), reproductive processes (*n* = 6, 10%) and multi-organism processes (*n* = 6, 10%). In the CC category, genes were enriched in cells (*n* = 35, 58.3%) and organelles (*n* = 35, 58.3%) (Figure 4A). The results for GO function enrichment showed that members of the plant *bHLH* gene family possess multiple functions and are important for plant resistance, growth, and development. KEGG enrichment analysis also indicated that the SabHLH family functions in various transduction pathways as TFs, and some members are involved in plant hormone signal transduction, environmental information processing, and genetic information processing (Figure 4B). These functions and processes are closely related to the main function of the SabHLH protein, which functions as a TF that regulates the expression of downstream genes.

### Spatial and Temporal Expression Patterns of *SabHLH* Genes

**FIGURE 5:**
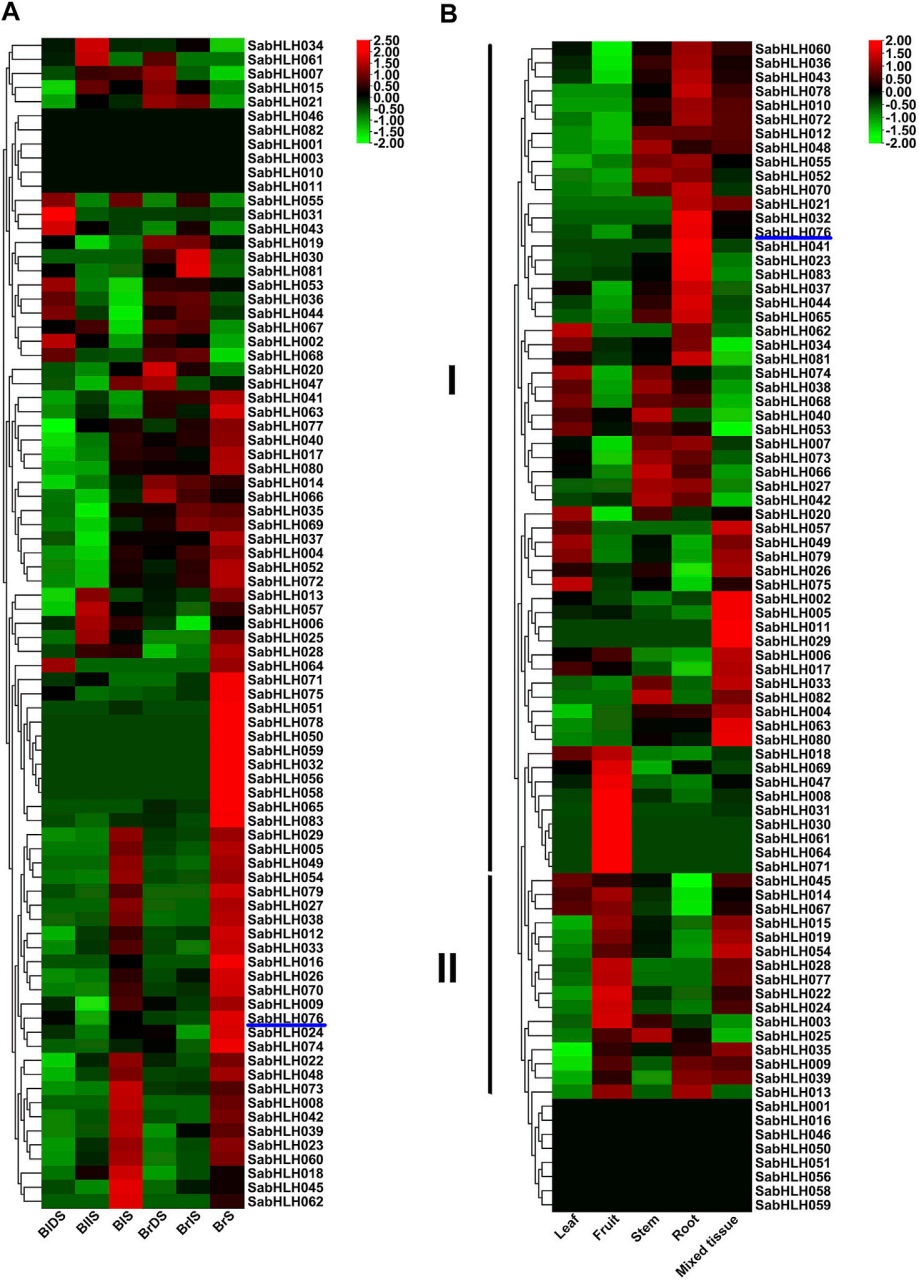
RNA-Seq expression analysis of *SabHLHs* obtained from the public transcriptomic database of *S. aralocaspica*. **(A)** Gene expression pattern of *SabHLHs* in different tissues. **(B)** Gene expression pattern of *SabHLHs* in dimorphic seeds during germination. The fragments per kilobase of exon per million mapped reads (FPKM) values from the RNA-Seq data were log_2_ transformed. BIDS, black dry seed; BIIS, black imbibed seed (imbibition for 24 h); BIS, seeding germinated from black seed (germination for 10 days); BrDS, brown dry seed; BrlS, brown imbibed seed (imbibition for 1 h); BrS, seeding germinated from brown seed (imbibition for 24 h). Roman numerals I and II represent different subfamilies. The color scale is shown at the right and higher expression levels are shown in red.

The expression patterns of *SabHLH* genes in different tissues (leaf, fruit, stem, root, and mixed tissue) and germination of dimorphic seeds (dry, imbibed, and germinated seeds) were analyzed based on available RNA-Seq data (Wang L. et al., 2017; Wang et al., 2019a). As shown in Figure 5A, a large number of *SabHLH* genes accumulated more transcripts during dimorphic seed germination compared to dry seeds, and the brown seedlings responded quicker and more highly than the black ones. Twenty-two *SabHLH* genes presented similar expression patterns in BlS and BrS, and half of these genes were highly expressed in seedlings germinated from brown (*SabHLH-032*, -*050*, *-051*, *-056*, *-058*, *-059*, *-065*, *-071*, *-075*, *-078,* and *-083*) and black (*SabHLH-008*, *-018*, *-022*, *-023*, *-039*, *-042*, *-045*, *-048*, *-060*, *-062,* and *-073*) seeds, respectively, suggesting that these genes may play distinct roles in dimorphic seed germination and seedling development. As shown in Figure 5B, among 83 *SabHLH* genes, four were specifically detected in mixed tissues, and eight were not expressed in any of the detected tissues. The majority of *SabHLH* genes presented different expression patterns, whereas a few exhibited similar expression profiles, and could be divided into two groups. Group I genes were expressed at relatively high levels in roots, steam, and leaves, and at low levels in fruits, whereas group II genes were expressed at high levels in fruits. In addition, several *SabHLHs* were expressed at very high levels in specific tissues. For example, eight genes (*SabHLH-008*, *-030*, *-031*, *-047*, *-061*, *-064*, *-069,* and *-071*) were expressed at the highest levels in fruit and may play specific roles in fruit development. Overall, most *SabHLH* genes were expressed in roots, and the number of expressed genes in different tissues followed the order root > fruit > stem > leaf.

### Expression Profiles of *SabHLH* Genes in Response to Abiotic Stresses

**FIGURE 6:**
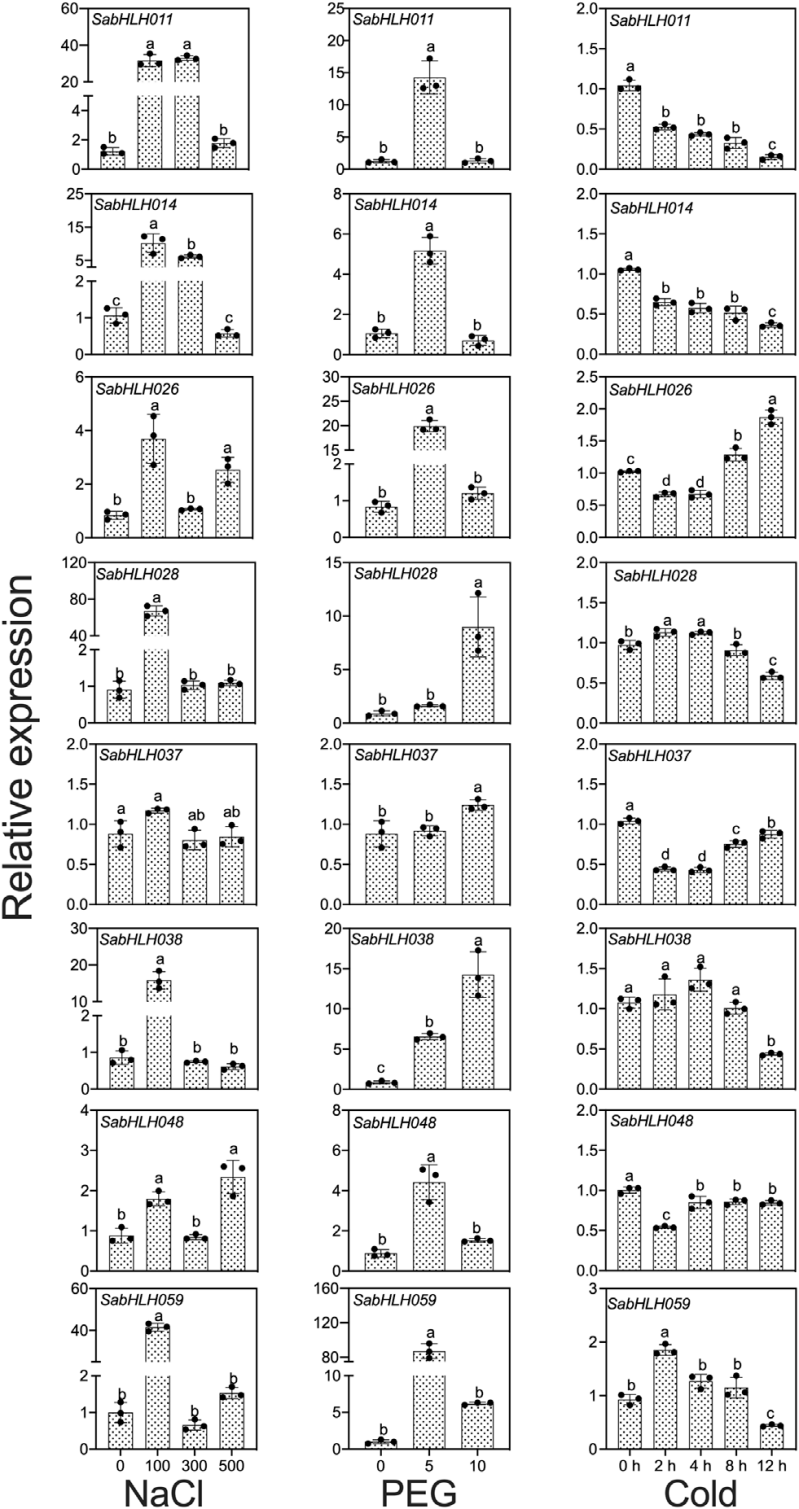
Gene expression of eight *SabHLH* candidate genes under different abiotic stresses at seedling stages based on qRT-PCR. 100, 300, and 500 mmol L^−1^ NaCl were used to simulate salt stress. 5% and 10% PEG were used to simulate drought stress. Cold stress was exposure to 4°C treatment for 0, 2, 4, 8 and 12 h. Different lowercase letters indicate a significant difference of the same gene at different treatments. Values are means ± SD of three biological replicates.

**FIGURE 7:**
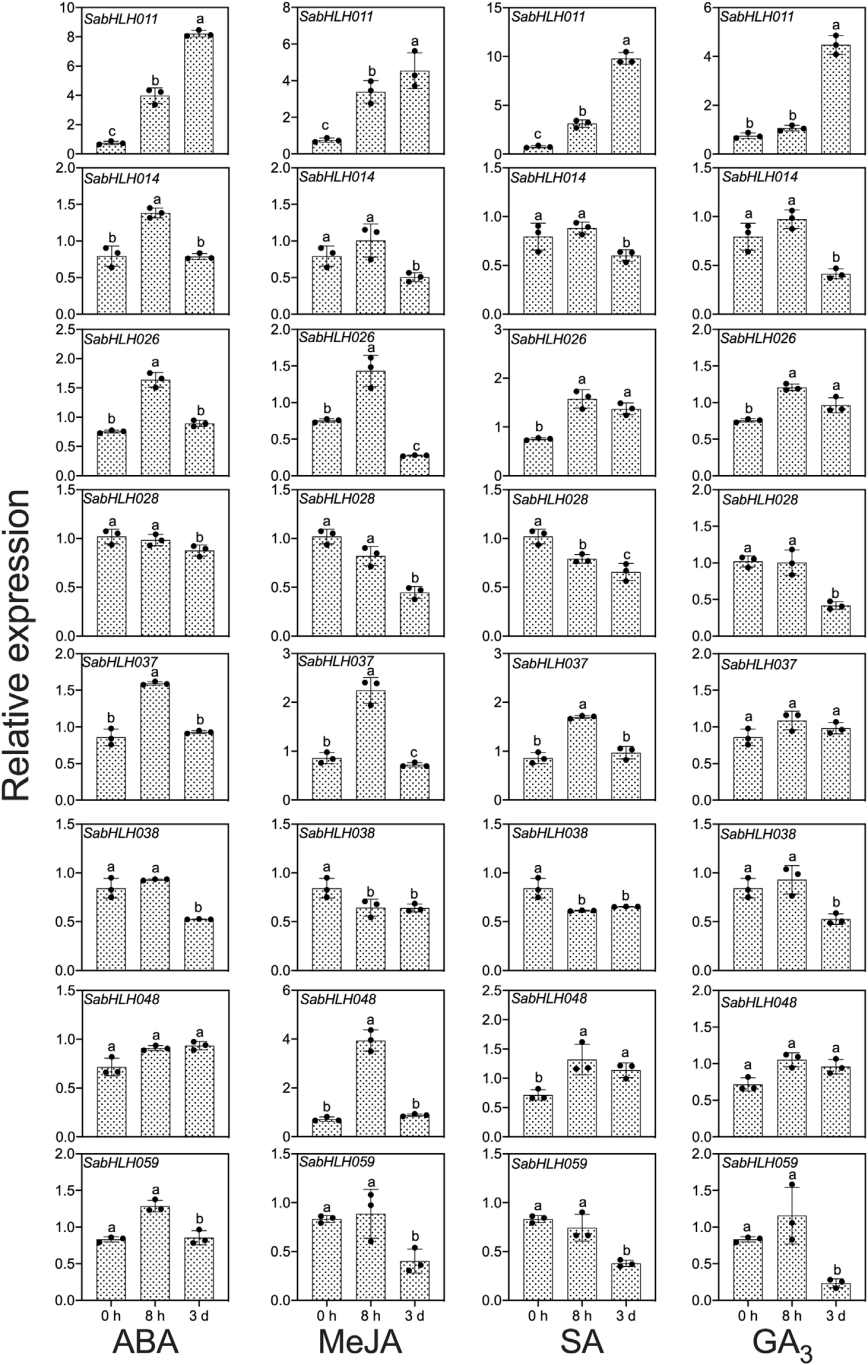
Gene expression of eight *SabHLH* candidate genes under different hormone treatments at seedling stage based on qRT-PCR. Treatment with 0.5 μmol L^−1^ abscisic acid (ABA), 0.5 μmol L^−1^ methyl jasmonate (MeJA), 1.5 mg mL^−1^ salicylic acid (SA), and 800 mg mL^−1^ gibberellic acid (GA_3_) for 0 h, 8 h, and 3 days, respectively. Different lowercase letters indicate a significant difference of the same gene at different treatments. Values are means ± SD of three biological replicates.

To investigate the responses of *SabHLHs* to NaCl, PEG, low temperature, and different phytohormones in developing seedlings, the transcriptional expression patterns of the eight *SabHLH* genes were analyzed by qRT-PCR. These genes were selected because they are highly expressed in different tissues and developing seedlings. The majority of these eight *SabHLH* genes were significantly upregulated with the increasing degree of salt and drought stress and downregulated under extended durations of cold treatment (Figure 6). In addition, high expression levels of most *SabHLHs* were observed under ABA, MeJA, and SA treatments, while some genes were downregulated under GA_3_ treatment (Figure 7).

After salt treatment, seven genes (*SabHLH-011*, *-014*, *-026*, *-028*, *-038*, *-048,* and *-059*) were more highly expressed under 100 mmol L^−1^ NaCl, while *SabHLH037* expression was not changed. The expression levels of five genes (*SabHLH011*, *-014*, *-028*, *-038,* and *-059*) were significantly increased by at least 20-fold. Under 300 mmol L^−1^ salt treatment, the expression of *SabHLH011* and *SabHLH014* increased 26.6- and 6-fold, respectively, while the expression of other genes did not change. Under high concentrations (500 mmol L^−1^) of salt, the relative expression levels of *SabHLH026* and *SabHLH048* increased by 3- and 2-fold, respectively (Figure 6).

The relative expression levels of *SabHLH028*, *SabHLH037* and *SabHLH038* were significantly upregulated under increasing concentrations of PEG. The relative expression levels of five genes (*SabHLH-011*, *-014*, *-026*, *-048,* and *-059*) showed a similar trend, being significantly increased under 5% PEG treatment, but downregulated under 10% PEG treatment. The expression of *SabHLH011*, *SabHLH014* and *SabHLH026* increased 11.6-, 5.5- and 24.5-fold, respectively; notably, *SabHLH059* gene expression peaked at an 80-fold increase. However, the relative expression levels of *SabHLH028* and *SabHLH037* increased under 10% PEG treatment but were not significantly different from the control. *SabHLH028* and *SabHLH037* were increased 11.1- and 1.3-fold, respectively (Figure 6).

After cold stress treatment, the expression levels of five genes (S*abHLH-011*, -*014*, -*037*, -*038,* and -*048*) were downregulated, while those of S*abHLH028* and *SabHLH059* were significantly upregulated and then downregulated, respectively, whereas the opposite was true for *SabHLH026*. The expression levels of *SabHLH028* and *SabHLH059* increased by 1.15- and 2.01-fold after 2 h of treatment (Figure 6).

Many *SabHLHs* responded to more than one hormone treatment in *S. aralocaspica*, and four genes were partially induced by the four hormone (ABA, GA_3_, MeJA, and SA) treatments. Interestingly, *SabHLH011* and *SabHLH026* were upregulated under all four hormone treatments, whereas *SabHLH028* and *SabHLH038* were downregulated (Figure 7). Four genes (*SabHLH-011*, *-014*, *-026,* and *-037*) were induced under ABA treatment (Figure 7), two genes (*SabHLH011* and *SabHLH026*) were induced under GA_3_ treatment (Figure 7), and four genes (*SabHLH-011, -026, -037,* and *-048*) were upregulated under MeJA and SA treatments (Figure 7). Notably, *SabHLH011* presented low or no expression in different tissues but was highly expressed following hormone treatment. Conversely, some genes that were normally highly expressed in different tissues were unresponsive to hormone treatment. Overall, most *SabHLH* genes responded to hormone treatments, indicating that this gene family plays important roles in hormone regulation in *S. aralocaspica*.

Among the eight *SabHLH* genes, *SabHLH011* was expressed at the highest level. Moreover, genes from the same clade presented similar expression patterns under certain hormonal treatments. For example, *SabHLH014* and *SabHLH028* from the III b subfamily followed similar expression patterns under ABA and GA_3_ treatments (Figure 7). However, their expression differed under ABA, MeJA and SA treatments, where *SabHLH059* was significantly downregulated and *SabHLH011* was significantly upregulated. In addition, analysis of *cis*-acting elements revealed that the promoter regions of these eight *SabHLHs* contained more than one *cis*-acting element related to the hormone response. Moreover, qRT-PCR analyses confirmed the hormone-induced expression characteristics of the eight *SabHLH* genes.

### The Expression Level, Subcellular Localization and Transcriptional Assay of SabHLH169(076)

**FIGURE 8:**
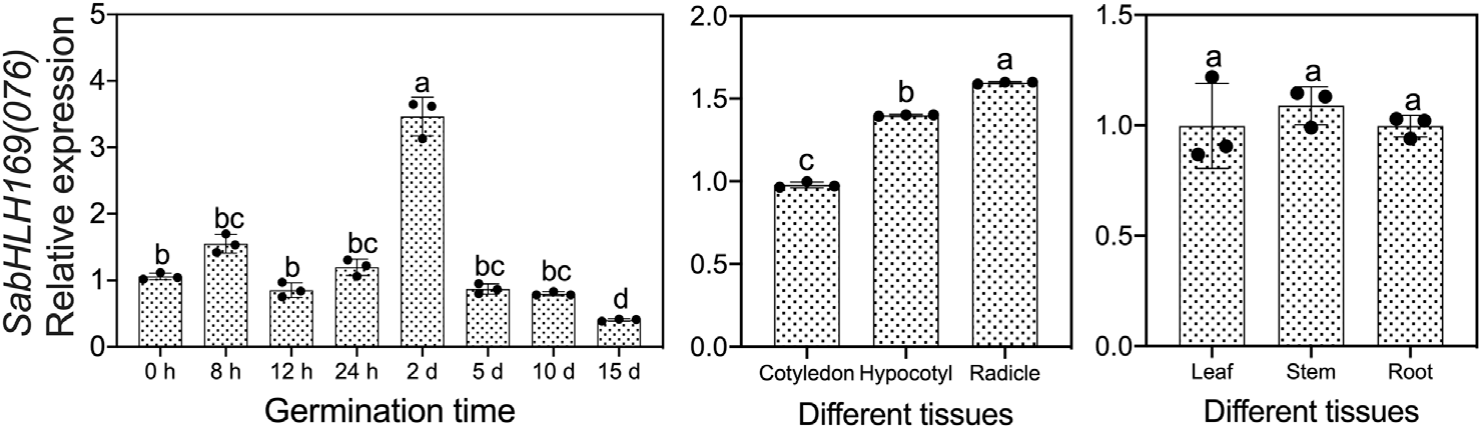
Spatial and temporal expression of *SabHLH169(076)* in seed germination and different tissues of *S. aralocaspica* based on qRT-PCR. Different lowercase letters indicate a significant difference at different treatments. Values are means ± SD of three biological replicates.

We previously screened the SabHLH169 (076) protein by DNA-pull down, but its function remains unknown, so we systematically explored its spatio-temporal expression pattern, its response to different light qualities, hormones, and abiotic stresses, and its subcellular localization and transcriptional self-activating activity. Based on the available RNA-Seq data, *SabHLH169(076)* accumulated more transcripts with the germination progression in brown seedlings and was preferentially expressed in roots (Figure 5). In the present study, the transcriptional expression level of *SabHLH169(076)* increased gradually from 0 to 8 h and was the highest at 2 days after germination. Meanwhile, the expression pattern of *SabHLH169(076)* in different tissues was consistent with the RNA-Seq data and was significantly detected in developing radicles (Figure 8).

**FIGURE 9:**
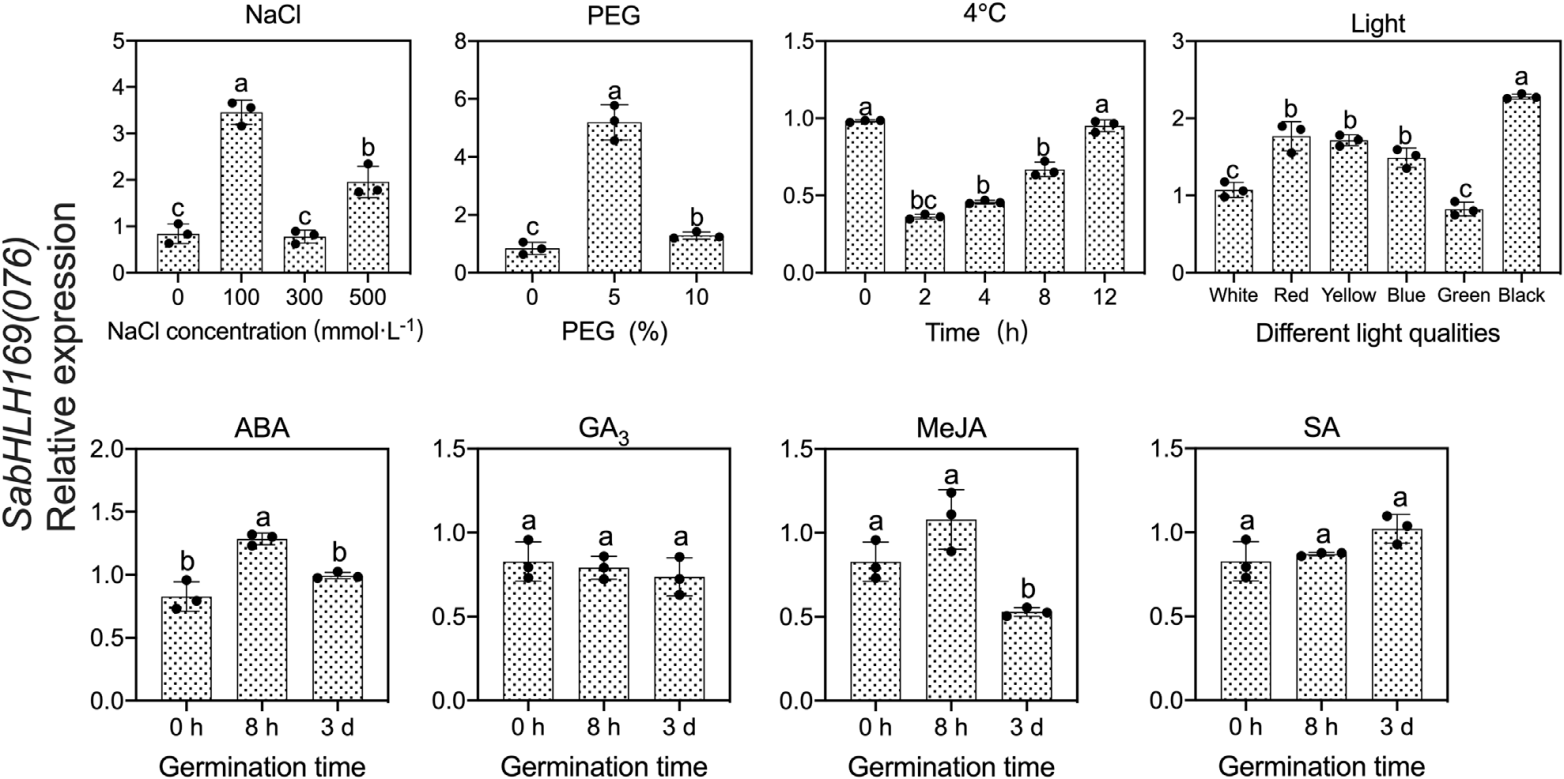
Gene expression of *SabHLH169(076)* under different light qualities and abiotic stresses based on qRT-PCR. Different lowercase letters indicate a significant difference at different treatments. Values are means ± SD of three biological replicates.

To investigate the response of *SabHLH169(076)* to salt, drought, cold stress, and different light qualities in developing seedlings, the expression patterns of *SabHLH169(076)* were analyzed (Figure 9). According to the results, *SabHLH169(076)* was significantly upregulated by low salt concentration (100 mmol L^−1^ NaCl) and drought stress (5% PEG), but downregulated by 4°C cold stress, decreasing significantly at 2 h and returning to control levels at 4–12 h after germination. Under different light qualities, *SabHLH169(076)* expression was the highest under dark conditions, followed by under red, yellow, and blue light. The expression was the lowest under white and green light, suggesting that *SabHLH169(076)* expression was significantly inhibited by white light. Under different hormone treatments, *SabHLH169(076)* expression significantly increased at 8 h but decreased at 3 days after germination when exposed to ABA treatment. No significant change in *SabHLH169(076)* expression was observed under the GA_3_, MeJA, and SA treatments. Overall, the trend of *SabHLH169(076)* expression at the transcriptional level was relatively moderate over the ranges of the tested hormone concentrations.

**FIGURE 10:**
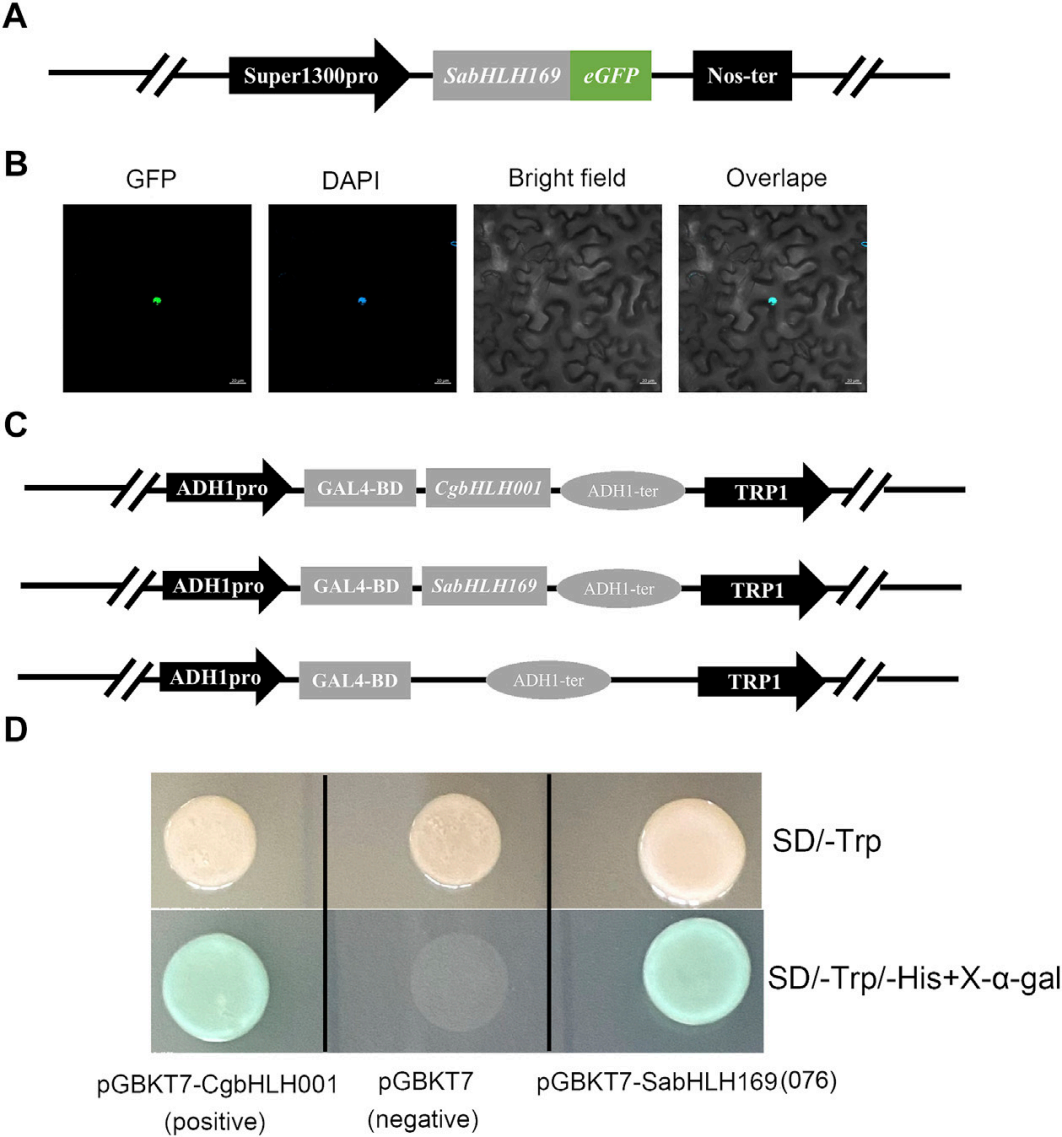
Subcellular localization and transcriptional activity analysis of SabHLH169(076) protein. **(A)** Schematic diagram of pSuper1300-*SabHLH169(076)*-eGFP vector construction. Super1300pro represents a superpromoter consisting of a trimer of the octopine synthase transcriptional activating element affixed to the mannopine synthase 2′ transcriptional activating element plus minimal promoter; eGFP represents enhanced green fluorescent protein; Nos-ter represents nopaline synthase terminator. **(B)** Subcellular localization of SabHLH169(076) protein. GFP, green fluorescent protein; DAPI, nuclear marker control. Bar = 20 µm. **(C)** Schematic diagram of pGBKT7-*CgbHLH001*, pGBKT7-*SabHLH169(076)* and pGBKT7 vector construction. **(D)** Transcriptional assay of SabHLH169(076). The transformed yeast cells were grown on SD/-Trp medium and add X-α-gal in SD/-His-Trp medium, respectively.

Prediction of *in silico* subcellular localization showed that approximately 98% SabHLH proteins were most likely localized in the nucleus (Table 1). A transient transformation assay in tobacco epidermal cells found a strong fluorescent signal for SabHLH169(076) in the nucleus, which is consistent with the prediction by the Plant-mPLoc software (Figure 10B). For the transactivation assay, the FL sequences of *SabHLH169(076)* and *CgbHLH001* (positive control) were fused to the vector pGBKT7 containing the GAL4 DNA-binding domain and subsequently transformed into yeast AH109. The yeast cells grew well on the selection media SD/-Trp and SD/-His-Trp/X-alpha-Gal and exhibited alpha-galactosidase activity. In contrast, AH109 containing the empty vector pGBKT7 (negative control), which lacks the transcriptional activation domain GAL4 AD, was unable to grow on the SD/-His-Trp medium. These results revealed that SabHLH169(076) possesses individual transcriptional activity in yeast (Figure 10D).

## Discussion

The bHLH TF family is one of the largest TF gene families in eukaryotic organisms, and regulates multiple aspects of plant growth, development, and stress responses (Riechmann et al., 2000). Therefore, it is necessary to identify the different *bHLH* isoforms and their expression characteristics to further understand their functions. The rapid development of plant genome sequencing technology has boosted the recent identification of *bHLH* gene families in at least 20 plant varieties. However, there have been few reports on desert halophytes. *S. aralocaspica*, a euhalophyte with a unique single-cell C_4_ (SCC_4_) pathway, has high photosynthetic efficiency and strong stress resistance in heterogeneous habitats (Smith et al., 2009; Sharpe and Offermann, 2014). Previously, we screened a putative SabHLH169 protein (named SabHLH076 in the present study) that may interact with the promoter of the *SaPEPC-1* gene (unpublished data), the key photosynthetic enzyme in *S. aralocaspica*; however, its biological function remains unknown. In the present study, we systemically characterized 83 *SabHLH* genes from the *S. aralocaspica* genome, which were clustered into 21 subfamilies. Members of different subfamilies showed large differences in protein sequence length, molecular weight, theoretical *pI* values and exon/intron structures; however, all were enriched in the nucleus based on the subcellular localization predictions, which is similar to the results in *Arabidopsis* and other plants (Carretero-Paulet et al., 2010). All SabHLHs contained motifs 1 and 2, the position of the bHLH domain that plays important roles in DNA binding and protein dimerization (Atchley and Fitch, 1997). Expression profiles derived from transcriptome data indicated that a large number of *SabHLH* genes were highly expressed in roots and fruits, and mainly detected in brown seed during post-germination growth. The expression levels of eight *SabHLH* genes were upregulated under abiotic stress and various hormone treatments, which may be partially related to the *cis*-elements distributed on the promoter. In addition, subcellular localization and transcriptional activity experiments showed that the SabHLH169(076) protein was mainly located in the nucleus and was self-activating, which further supports its functions as a TF that is involved in responses to light quality, drought and salt stress. Our results suggest that SabHLH may play an important role in improving the ability of *S. aralocaspica* to resist abiotic stresses during growth and development.

The number of *bHLH* gene family members is around 100–200 in the vast majority of species (Li et al., 2006; Zhang et al., 2018; Wang et al., 2019b), with other species having notable quantities, such as peach (95) (Zhang et al., 2018) and soybean (319) (Hudson and Hudson, 2015); relatively few (83) *bHLH* superfamily genes were identified in *S. aralocaspica* in the present study. This finding may be associated with differences in evolution and genome duplication or genome sizes in the plants. Gene duplication is considered one of the primary drivers of gene family expansion in plants and plays an important role in the evolution of new gene functions and adaptation (Flagel and Wendel, 2009). However, a decrease in the number of genes can also produce important genetic variation, which can in turn have positive effects on plant survival and reproduction. The current “less is more” hypothesis proposes that gene-number reduction events such as pseudogenization or loss of genes are as important as increase events (Yong et al., 2019). Owing to the imperfect state of the current genomic data, further covariance analysis and chromosomal localization of the gene cannot be performed to clarify the evolutionary relationships and localization of the *SabHLH* gene family. Perhaps the hypothesis can explain why the number of members of the *bHLH* gene family in *S. aralocaspica* is low, possibly as a result of selective evolution of *S. aralocaspica* to adapt to external conditions. The distributions of introns, exons and UTRs vary widely among various *bHLH* genes. There are 365 exons in the *S. aralocaspica bHLH* gene family, and 193 exons are symmetric. Among the 371 introns found in the *SabHLH* genes, 319 were in phase 0, 38 were in phase 1, and 14 were in phase 2. Exons with the same splicing phase at both ends are termed symmetric exons, and an excess of symmetric exons and phase 0 introns is likely to facilitate exon shuffling, recombinational fusion, and protein domain exchange (Gilbert, 1987; Patthy, 1987). Therefore, the analysis of the *bHLH* gene structures in *S. aralocaspica* indicated a large diversity of bHLH TFs, which has also been reported in *Salvia miltiorrhiza* (Zhang et al., 2015) and *Vitis vinifera* (Wang et al., 2018).

Currently, there is no specific classification for grouping the plant *bHLH* gene family, and the subgroups contained in the *bHLH* gene families of different species vary. The *Arabidopsis bHLH* gene family contains 15 clades and some orphans (Toledo-Ortiz et al., 2003), rice contains 22 clades (Li et al., 2006), grape contains 15 clades (Wang et al., 2018), and apple contains 18 clades (Ma et al., 2017). These subfamilies are common in most species, suggesting that bHLH proteins in conserved subfamilies might play an important role in plant evolution. The *S. aralocaspica bHLH* gene family contains 21 clades (Figure 1), with subgroup X containing 10 AtbHLHs, but no SabHLHs*,* indicating that the species is evolving in multiple directions. Non-conserved bHLH subfamilies among certain plant species may have evolved to meet the developmental needs of plants or in response to stress (Zhang et al., 2020). Considering that approximately 40% of *Arabidopsis* bHLH proteins have been functionally characterized (Sun et al., 2015; Wang et al., 2018). The clustering and comparison of SabHLH proteins with AtbHLHs can facilitate the prediction of their functions via ortholog analysis. In the present study, there were 16 SabHLHs tightly grouped with the AtbHLH, in which 11 AtbHLH functions were known. For example, AtFIT (AtbHLH029) was the essential protein involved in iron uptake responses (Colangelo and Guerinot, 2004), and AtFAMA (AtbHLH097) could interact with AtSPCH and AtMUTE (AtbHLH045) to regulate stomata formation (Ohashi-Ito and Bergmann, 2006). Therefore, the corresponding SabHLH065, SabHLH079 and SabHLH021 may perform functions similar to those of their *Arabidopsis* orthologs (Supplementary Table S3).

Patterns of gene expression are important for determining the function and characteristics of the *bHLH* gene family. In the present study, the majority of *SabHLH* genes were activated in dimorphic seeds and more transcripts were accumulated with the progression of brown seed germination (Figure 5A). According to Wang L. et al. (2017), during the germination process of dimorphic seeds of *S. aralocaspica*, the secondary metabolism (flavonoid and flavonol biosynthesis) pathway was activated earlier in brown seed compared with in black seed, with the seeds showing different germination behaviors in order to cope with the harsh and unpredictable environment (Guo W. L. et al., 2020). In sheepgrass (*Leymus chinensis*), *LcbHLH92* negatively regulates the accumulation of anthocyanins, with effects on seed coat color and reduction of seed dormancy (Zhao et al., 2019). The yellow seed sheepgrass germplasm was found to possess lower anthocyanidin contents and germinated more quickly compared with brown seeds. *In S. aralocaspica*, PIF (SabHLH026), was expressed at the highest level in Brs and Bls, which helps seeds to break dormancy and regulate germination during seed development (Wang L. et al., 2017), which is also observed in sheepgrass (Li et al., 2019).

Through KEGG analysis, we observed that SabHLHs also participated in plant hormone signal transduction pathways, so that it may also influence the process of seed germination. ABA and GA_3_ are key endogenous signaling molecules involved in seed dormancy acquisition or release. Low ABA concentration promoted seed germination and seedling growth in *S. aralocaspica* (Cao et al., 2015). Consistently, in the present study, exogenous ABA and GA_3_ treatment significantly upregulated the expression of *SabHLH* genes (Figure 7). Similar to the functions of bHLH in *Brassica napus*, *SabHLH* was highly expressed in root, which may mainly be involved in the regulation of root development, salt and drought stress response, and hormone responses. ABA plays an important role in plant responses to abiotic stresses, such as low temperature, drought, and salinity. In *Arabidopsis*, *AtbHLH17* (*AIB*) positively responds to NaCl and mannitol stress, *AtbHLH129* regulates root elongation and the ABA response, and *AtbHLH006* (*MYC2*) and *AtbHLH112* are involved in root growth and tolerance to salt stress (Gupta et al., 2014; Tian et al., 2015; Chen et al., 2017; Du et al., 2018; Peñuelas et al., 2019). bHLH TF *AtPRE6* is involved in the ABA-mediated regulation of salt response, and *AtPRE6* gene expression levels are reduced in response to ABA treatment but increased during salt treatment (Zheng et al., 2019). In the present study, *SabHLH028*, *SabHLH038* and *SabHLH059* exhibited similar trends under ABA and salt treatment (Figures 6, 7), suggesting that they have similar functions in responses to ABA and salt stress responses. Salicylic acid, a simple phenolic compound existing widely in higher plants, not only regulates plant growth and metabolism, but also plays a leading role in plant immunity against disease and environmental stress, such as salt, cold, and heavy metal stress (Zhang et al., 2016). In the present study, only two out of the eight candidate genes were upregulated after exogenous SA treatment, and most of them were down-regulated or non-responsive (Figure 7). In *Salvia miltiorrhiza*, a total of 99 *SmbHLH* genes were found to respond to SA, but only three were upregulated and 12 were downregulated (Zhang et al., 2016). In the present study, *SabHLH037* and *SabHLH169(076)* highly expressed in roots, were significantly upregulated by ABA, MeJA, and SA treatments. This indicated that the *SabHLH* genes may play important roles in seed dormancy, germination, root development and hormone signal transduction.

The regulation of gene expression via specific *cis*-regulatory elements in promoter regions has evolved as a major adaptive mechanism in the response of plants to environmental conditions (Walther et al., 2007). *Cis*-element analyses revealed a wide range of stress-responsive elements in the promoters of *SabHLH* genes (Supplementary Figure S1). For example, the promoter regions of *SabHLH011*, *SabHLH028* and *SabHLH037* contained 2, 2 and 1 MBS elements (MYB binding sites involved in drought-inducibility), respectively, leading to significantly high expression of these genes under drought stress treatments (Figure 6). This is consistent with the findings of a previous study on pepper bHLH (Liu et al., 2021). *Arabidopsis AtUNE12* belongs to the bHLH TF superfamily, which can be induced by NaCl, mannitol and ABA to confer salt and osmotic stress tolerance in plants (He et al., 2021). In the present study, the expression levels of eight candidate *SabHLH* genes were all increased under NaCl treatment, especially *SabHLH038* (homologous to *AtUNE12*) was significantly upregulated under both NaCl and PEG treatments, and may play a role similar to that of *AtUNE12* in *S. aralocaspica*. *SabHLH* gene promoters have been observed to harbor 63 LTR elements, indicating that they may be regulated by low temperature (Xu et al., 2014). The CBF cold response pathway plays a central role in cold acclimation (Thomashow, 2001). *ICE*, a member of the bHLH family, can directly interact with CBF protein to enhance plant tolerance to low temperature (Zarka et al., 2003). In addition, *OsICE1* and *OsICE2* overexpression significantly enhanced the cold tolerance of *Arabidopsis* seedlings and improved the expression of cold-response genes (Deng et al., 2017). In the present study, we identified two *ICE* genes, *SabHLH014* and *SabHLH028*, which positively responded to PEG and NaCl treatments. Under low-temperature stress, *SabHLH014* expression tended to decrease, while *SabHLH028* expression tended to increase for 2–4 h. Studies have shown that within 6 h of cold stress, upregulated early cold-response genes are mainly associated with transcription and cell signal transduction, while the 24 h cold-response genes are mostly related to gene transcription and metabolic activities (Lee et al., 2005). There were abundant ABA-, MeJA-, GA_3_-, and SA-responsive elements in the promoter sequences of *SabHLH* genes, suggesting that these genes may be involved in the transcriptional control of hormone responses. For example, the *SabHLH026* gene, which contains ABA (ABRE) and MeJA (CGTCA-motif) responsive elements in its promoter regions, was significantly upregulated by exogenous hormone treatment. Similarly, the promoter region of *SabHLH037* contains the SA-responsive element (TCA-motif), which was significantly upregulated by exogenous SA hormone treatment (Figure 7). Similar results have been found for grapes, and the promoters of *VvbHLH* genes contained ABRE and DRE elements, which are involved in ABA-dependent or ABA-independent stress tolerance (Wang et al., 2018). In addition, most *SabHLH* gene promoters contain G-box elements, indicating that they may be regulated by other *S. aralocaspica* bHLHs and may form a regulatory network that responds to different stresses. This is consistent with our predicted results for the bHLH protein interaction network in *S. aralocaspica* (Figure 3).

bHLH TFs are not only universally involved in plant response to stress, but also play an important role in light signal transduction and photomorphogenesis. Among them, the best characterized is the PIF families of bHLH TFs that act mainly as negative regulators of photosynthesis gene expression in response to light availability (Pham et al., 2018). *SabHLH169(076)* was the first *bHLH* gene cloned in *S. aralocaspica,* in addition to positively responding to salt, drought and cold stress, we found that the relative expression of *SabHLH169(076)* was much higher in darkness than in light (any color), especially normal light (Figure 8), which is similar to the results of *ZmbHLH80* and *ZmbHLH90* identified in maize (Górska et al., 2019, 2021). *ZmbHLH80* and *ZmbHLH90* had the same expression profiles with a peak at night or at the beginning of the day and a decline after dawn until the end of the photoperiod. Moreover, the transcript levels of *ZmbHLH80* and *ZmbHLH90* were higher in roots, stems, and etiolated leaves than in green leaves, which is consistent with the results of the present study. *SabHLH169(076)* was expressed in all tissues at the germination stage with expression levels in the order of radicle > hypocotyl > cotyledon. These results suggest that *SabHLH169(076)* may be negatively regulated by light and preferentially expressed in cells and tissues with lower photosynthetic activity.

## Conclusions

To the best of our knowledge, this is the first comprehensive and systematic genome-wide analysis of the *S. aralocaspica bHLH* superfamily. In the present study, 83 *SabHLH* genes were identified and the divergent biochemical characteristics of SabHLH proteins were analyzed. Based on the results of conserved motif and intron-exon organization and phylogenetic analyses, the SabHLH family was classified into 21 groups. Protein association network predictions and functional classification analysis revealed multiple functions of the SabHLH proteins. *Cis*-elements analysis revealed that *SabHLH* contains many promoter elements related to hormone and stress responses. RNA-seq and qRT-PCR analyses illustrated that *SabHLH* genes are expressed in dimorphic seeds during germination and in different tissues, and respond to different abiotic stresses at the transcriptional level. SabHLH169(076) is localized in the nucleus with transcriptional self-activating activity and may function as a TF to regulate numerous physiological processes. Overall, these data provide a reference for further studies on the abiotic stress resistance mechanisms of the *bHLH* gene in *S. aralocaspica*.

## Data Availability

The original contributions presented in the study are included in the article/Supplementary Material, further inquiries can be directed to the corresponding author.

## Supplementary Material

Supplementary Figure S1 Predicted *cis*-elements on promoters of 83 *bHLH* genes in *S. aralocaspica*. The promoter regions (2,000 bp) of *SabHLH* genes were analyzed using PlantCARE software. Different colored boxes indicate the *cis*-elements related to growth, development, plant hormones, and stresses.

## References

[B1] Atchley W. R. Fitch W. M. (1997). A Natural Classification of the Basic Helix-Loop-Helix Class of Transcription Factors. Proc. Natl. Acad. Sci. U.S.A. 94, 5172–5176. 10.1073/pnas.94.10.5172 9144210 PMC24651

[B2] Atchley W. R. Terhalle W. Dress A. (1999). Positional Dependence, Cliques, and Predictive Motifs in the bHLH Protein Domain. J. Mol. Evol. 48, 501–516. 10.1007/PL00006494 10198117

[B3] Bu Q. Castillon A. Chen F. Zhu L. Huq E. (2011). Dimerization and Blue Light Regulation of *PIF1* Interacting bHLH Proteins in *Arabidopsis* . Plant Mol. Biol. 77, 501–511. 10.1007/s11103-011-9827-4 21928113

[B4] Buti S. Pantazopoulou C. K. van Gelderen K. Hoogers V. Reinen E. Pierik R. (2020). A Gas-and-Brake Mechanism of bHLH Proteins Modulates Shade Avoidance. Plant Physiol. 184, 2137–2153. 10.1101/2020.05.07.08273510.1104/pp.20.00677 33051265 PMC7723099

[B5] Cao J. Cheng G. Wang L. Maimaitijiang T. Lan H. (2021). Genome-Wide Identification and Analysis of the Phosphoenolpyruvate Carboxylase Gene Family in *Suaeda aralocaspica*, an Annual Halophyte with Single-Cellular C_4_ Anatomy. Front. Plant Sci. 12, 665279. 10.3389/fpls.2021.665279 34527003 PMC8435749

[B100] Cao J. Li X. Wang C. Wang L. Lan X. Lan H. (2015). Effects of Exogenous Abscisic Acid on Heteromorphic Seed Germination of *Suaeda aralocaspica*, a Typical Halophyte of Xinjiang Desert Region (in Chinese). Acta Ecol. Sin. 35, 6666–6677. 10.5846/stxb201405130978

[B6] Cao J. Li X. R. Chen L. He M. X. Lan H. Y. (2022). The Developmental Delay of Seedlings with Cotyledons Only Confers Stress Tolerance to *Suaeda aralocaspica* (Chenopodiaceae) by Unique Performance on Morphology, Physiology and Gene Expression. Front. Plant Sci. 13, 844430. 10.3389/fpls.2022.844430 PMC9208309 35734249

[B7] Cao J. Wang L. Lan H. (2016). Validation of Reference Genes for Quantitative RT-PCR Normalization in *Suaeda aralocaspica*, an Annual Halophyte with Heteromorphism and C_4_ Pathway without Kranz Anatomy. Peer J. 4, e1697. 10.7717/peerj.1697 26893974 PMC4756755

[B8] Carretero-Paulet L. Galstyan A. Roig-Villanova I. Martínez-García J. F. Bilbao-Castro J. R. Robertson D. L. (2010). Genome-Wide Classification and Evolutionary Analysis of the bHLH Family of Transcription Factors in Arabidopsis, Poplar, Rice, Moss, and Algae. Plant Physiol. 153, 1398–1412. 10.1104/pp.110.153593 20472752 PMC2899937

[B9] Chen C. Chen H. Zhang Y. Thomas H. R. Frank M. H. He Y. (2020). Tbtools: an Integrative Toolkit Developed for Interactive Analyses of Big Biological Data. Mol. Plant 13, 1194–1202. 10.1016/j.molp.2020.06.009 32585190

[B10] Chen H. C. Hsieh-Feng V. Liao P. C. Cheng W. H. Liu L. Y. Yang Y. W. (2017). The Function of *OsbHLH068* Is Partially Redundant with its Homolog, *AtbHLH112*, in the Regulation of the Salt Stress Response but Has Opposite Functions to Control Flowering in *Arabidopsis* . Plant Mol. Biol. 94, 531–548. 10.1007/s11103-017-0624-6 28631168 PMC5504132

[B11] Cheng G. Wang L. Lan H. (2016). Cloning of *PEPC-1* from a C_4_ Halophyte *Suaeda aralocaspica* without Kranz Anatomy and its Recombinant Enzymatic Activity in Responses to Abiotic Stresses. Enzyme Microb. Technol. 83, 57–67. 10.1016/j.enzmictec.2015.11.006 26777251

[B12] Colangelo E. P. Guerinot M. L. (2004). The Essential Basic Helix-Loop-Helix Protein FIT1 Is Required for the Iron Deficiency Response. Plant Cell. 16, 3400–3412. 10.1105/tpc.104.024315 15539473 PMC535881

[B13] Commissione Redactorum Florae Xinjiangensis (1994). Flora Xinjiangensis. Urumchi: Xinjiang Science & Technology & Hygiene Publishing House.

[B14] Marcos A. D. Houbaert A. Triviño M. Delgado D. Martín-Trillo M. Russinova E. (2017). A Mutation in the bHLH Domain of the SPCH Transcription Factor Uncovers a BR-dependent Mechanism for Stomatal Development. Plant Physiol. 174, 823–842. 10.1104/pp.17.00615 28507175 PMC5462054

[B15] Deng C. Ye H. Fan M. Pu T. Yan J. (2017). The Rice Transcription Factors *OsICE* Confer Enhanced Cold Tolerance in Transgenic Arabidopsis. Plant Signal. Behav. 12, e1316442. 10.1080/15592324.2017.1316442 28414264 PMC5501220

[B16] Du T. Niu J. Su J. Li S. Guo X. Li L. (2018). SmbHLH37 Functions Antagonistically with SmMYC2 in Regulating Jasmonate-Mediated Biosynthesis of Phenolic Acids in *Salvia miltiorrhiza* . Front. Plant Sci. 9, 1720. 10.3389/fpls.2018.01720 30524467 PMC6262058

[B17] Flagel L. E. Wendel J. F. (2009). Gene Duplication and Evolutionary Novelty in Plants. New Phytol. 183, 557–564. 10.1111/j.1469-8137.2009.02923.x 19555435

[B18] Gao F. Robe K. Gaymard F. Izquierdo E. Dubos C. (2019). The Transcriptional Control of Iron Homeostasis in Plants: a Tale of bHLH Transcription Factors? Front. Plant Sci. 10, 6. 10.3389/fpls.2019.00006 30713541 PMC6345679

[B19] Gilbert W. (1987). The Exon Theory of Genes. Cold Spring Harb. Symposia quantitative Biol. 52, 901–905. 10.1101/SQB.1987.052.01.098 2456887

[B20] Górska A. M. Gouveia P. Borba A. R. Zimmermann A. Serra T. S. Carvalho P. (2021). ZmOrphan94 Transcription Factor Downregulates *ZmPEPC1* Gene Expression in Maize Bundle Sheath Cells. Front. Plant Sci. 12, 246. 10.3389/fpls.2021.559967 PMC8062929 33897718

[B21] Górska A. M. Gouveia P. Borba A. R. Zimmermann A. Serra T. S. Lourenço T. F. (2019). ZmbHLH80 and ZmbHLH90 Transcription Factors Act Antagonistically and Contribute to Regulate *PEPC1* Cell-specific Gene Expression. Plant J. 99, 270–285. 10.1111/tpj.14323 30900785

[B22] Gremski K. Ditta G. Yanofsky M. F. (2007). The *HECATE* Genes Regulate Female Reproductive Tract Development in *Arabidopsis thaliana* . Development 134, 3593–3601. 10.1242/dev.011510 17855426

[B23] Groszmann M. Bylstra Y. Lampugnani E. R. Smyth D. R. (2010). Regulation of Tissue-specific Expression of *SPATULA*, a *bHLH* Gene Involved in Carpel Development, Seedling Germination, and Lateral Organ Growth in *Arabidopsis* . J. Exp. Bot. 61, 1495–1508. 10.1093/jxb/erq015 20176890 PMC2837263

[B24] Guo J. Liu L. Du M. Tian H. Wang B. (2020). Cation and Zn Accumulation in Brown Seeds of the Euhalophyte *Suaeda salsa* Improves Germination under Saline Conditions. Front. Plant Sci. 11, 602427. 10.3389/fpls.2020.602427 33381136 PMC7767863

[B25] Guo W. L. Chen B. H. Guo Y. Y. Chen X. J. Li Q. F. Yang H. L. (2020). Expression of Pumpkin *CmbHLH87* Gene Improves Powdery Mildew Resistance in Tobacco. Front. Plant Sci. 11, 163. 10.3389/fpls.2020.00163 32318077 PMC7147351

[B26] Gupta N. Prasad V. B. R. Chattopadhyay S. (2014). *LeMYC2* Acts as a Negative Regulator of Blue Light Mediated Photomorphogenic Growth, and Promotes the Growth of Adult Tomato Plants. BMC Plant Biol. 14, 38. 10.1186/1471-2229-14-38 24483714 PMC3922655

[B27] He Z. Wang Z. Nie X. Qu M. Zhao H. Ji X. (2021). Unfertilized Embryo Sac 12 Phosphorylation Plays A Crucial Role in Conferring Salt Tolerance. Plant Physiol. 188, 1385–1401. 10.1093/plphys/kiab549 PMC8825338 34904673

[B28] Holsters M. De Waele D. Depicker A. Messens E. Van Montagu M. Schell J. (1978). Transfection and Transformation of Agrobacterium Tumefaciens. Molec. Gen. Genet. 163, 181–187. 10.1007/BF00267408 355847

[B29] Hudson K. A. Hudson M. E. (2015). A Classification of Basic Helix-Loop-Helix Transcription Factors of Soybean. Int. J. Genomics 2015, 1–10. 10.1155/2015/603182 PMC4339708 25763382

[B30] Hyun Y. Lee I. (2006). KIDARI, Encoding a Non-DNA Binding bHLH Protein, Represses Light Signal Transduction in *Arabidopsis thaliana* . Plant Mol. Biol. 61, 283–296. 10.1007/s11103-006-0010-2 16786307

[B31] Kanaoka M. M. Pillitteri L. J. Fujii H. Yoshida Y. Bogenschutz N. L. Takabayashi J. (2008). *SCREAM/ICE1* and *SCREAM2* Specify Three Cell-State Transitional Steps Leading to Arabidopsis Stomatal Differentiation. Plant Cell. 20, 1775–1785. 10.1105/tpc.108.060848 18641265 PMC2518248

[B32] Ke Y. Z. Wu Y. W. Zhou H. J. Chen P. Wang M. M. Liu M. M. (2020). Genome-wide Survey of the *bHLH* Super Gene Family in *Brassica napus* . BMC Plant Biol. 20, 115. 10.1186/s12870-020-2315-8 32171243 PMC7071649

[B33] Kondou Y. Nakazawa M. Kawashima M. Ichikawa T. Yoshizumi T. Suzuki K. (2008). Retarded Growth of Embryo1, a New Basic Helix-Loop-Helix Protein, Expresses in Endosperm to Control Embryo Growth. Plant Physiol. 147, 1924–1935. 10.1104/pp.108.118364 18567831 PMC2492639

[B34] Kumar S. Stecher G. Li M. Knyaz C. Tamura K. (2018). MEGA X: Molecular Evolutionary Genetics Analysis across Computing Platforms. Mol. Biol. Evol. 35, 1547–1549. 10.1093/molbev/msy096 29722887 PMC5967553

[B35] Kumar S. V. Lucyshyn D. Jaeger K. E. Alós E. Alvey E. Harberd N. P. (2012). Transcription Factor PIF4 Controls the Thermosensory Activation of Flowering. Nature 484, 242–245. 10.1038/nature10928 22437497 PMC4972390

[B36] Ledent V. Vervoort M. (2001). The Basic Helix-Loop-Helix Protein Family: Comparative Genomics and Phylogenetic Analysis. Genome Res. 11, 754–770. 10.1101/gr.177001 11337472 PMC311049

[B37] Lee S. C. Lee M. Y. Kim S. J. Jun S. H. An G. Kim S. R. (2005). Characterization of an Abiotic Stress-Inducible Dehydrin Gene, *OsDhn1*, in Rice (*Oryza sativa* L.). Mol. Cells 19, 212–218. 15879704

[B38] Li X. Duan X. Jiang H. Sun Y. Tang Y. Yuan Z. (2006). Genome-wide Analysis of Basic/helix-Loop-Helix Transcription Factor Family in Rice and *Arabidopsis* . Plant Physiol. 141, 1167–1184. 10.1104/pp.106.080580 16896230 PMC1533929

[B39] Li X. Liu S. Yuan G. Zhao P. Yang W. Jia J. (2019). Comparative Transcriptome Analysis Provides Insights into the Distinct Germination in Sheepgrass (*Leymus chinensis*) during Seed Development. Plant Physiology Biochem. 139, 446–458. 10.1016/j.plaphy.2019.04.007 30999132

[B40] Liu R. Song J. Liu S. Chen C. Zhang S. Wang J. (2021). Genome-wide Identification of the *Capsicum* bHLH Transcription Factor Family: Discovery of a Candidate Regulator Involved in the Regulation of Species-specific Bioactive Metabolites. BMC Plant Biol. 21, 1–18. 10.1186/s12870-021-03004-7 34098881 PMC8183072

[B41] Liu Y. Maimaitijiang T. Zhang J. Ma Y. Lan H. (2020). The Developmental Enhancement of a C_4_ System with Non-typical C_4_ Physiological Characteristics in *Salsola ferganica* (Kranz Anatomy), an Annual Desert Halophyte. Front. Plant Sci. 11, 152. 10.3389/fpls.2020.00152 32210984 PMC7069449

[B42] Long T. A. Tsukagoshi H. Busch W. Lahner B. Salt D. E. Benfey P. N. (2010). The bHLH Transcription Factor *POPEYE* Regulates Response to Iron Deficiency inArabidopsisRoots. Plant Cell. 22, 2219–2236. 10.1105/tpc.110.074096 20675571 PMC2929094

[B43] Lu R. Zhang J. Liu D. Wei Y. L. Wang Y. Li X. B. (2018). Characterization of *bHLH*/*HLH* Genes that Are Involved in Brassinosteroid (BR) Signaling in Fiber Development of Cotton (*Gossypium hirsutum*). BMC Plant Biol. 18, 1–13. 10.1186/s12870-018-1523-y 30482177 PMC6258498

[B44] Mao K. Dong Q. Li C. Liu C. Ma F. (2017). Genome Wide Identification and Characterization of Apple bHLH Transcription Factors and Expression Analysis in Response to Drought and Salt Stress. Front. Plant Sci. 8, 480. 10.3389/fpls.2017.00480 28443104 PMC5387082

[B45] Massari M. E. Murre C. (2000). Helix-loop-helix Proteins: Regulators of Transcription in Eucaryotic Organisms. Mol. Cell. Biol. 20, 429–440. 10.1128/MCB.20.2.429-440.2000 10611221 PMC85097

[B46] McDonald J. H. (2014). Handbool of Biological Statistics. 3rd Edn. Baltimore, Maryland: Sparky House Publishing.

[B47] Montero O. Velasco M. Sanz-Arranz A. Rull F. (2016). Effect of Different Broad Waveband Lights on Membrane Lipids of a Cyanobacterium, *Synechococcus* sp., as Determined by UPLC-QToF-MS and Vibrational Spectroscopy. Biology 5 (2), 22. 10.3390/biology5020022 PMC4929536 27223306

[B48] Ogo Y. Nakanishi Itai R. Nakanishi H. Kobayashi T. Takahashi M. Mori S. (2007). The Rice bHLH Protein OsIRO2 Is an Essential Regulator of the Genes Involved in Fe Uptake under Fe-Deficient Conditions. Plant J. 51, 366–377. 10.1111/j.1365-313X.2007.03149.x 17559517

[B49] Ohashi-Ito K. Bergmann D. C. (2006). *Arabidopsis FAMA* Controls the Final Proliferation/differentiation Switch during Stomatal Development. Plant Cell. 18, 2493–2505. 10.1105/tpc.106.046136 17088607 PMC1626605

[B50] Ohashi-Ito K. Bergmann D. C. (2007). Regulation of the Arabidopsis Root Vascular Initial Population by *LONESOME HIGHWAY* . Development 134, 2959–2968. 10.1242/dev.006296 17626058 PMC3145339

[B51] Pagnussat G. C. Yu H.-J. Ngo Q. A. Rajani S. Mayalagu S. Johnson C. S. (2005). Genetic and Molecular Identification of Genes Required for Female Gametophyte Development and Function in *Arabidopsis* . Development 132, 603–614. 10.1242/dev.01595 15634699

[B52] Pan C. Yang D. Zhao X. Liu Y. Li M. Ye L. (2021). *PIF4* Negatively Modulates Cold Tolerance in Tomato Anthers via Temperature-dependent Regulation of Tapetal Cell Death. Plant Cell. 33, 2320–2339. 10.1093/plcell/koab120 34009394 PMC8364245

[B53] Patthy L. (1987). Intron-dependent Evolution: Preferred Types of Exons and Introns. FEBS Lett. 214, 1–7. 10.1016/0014-5793(87)80002-9 3552723

[B54] Peñuelas M. Monte I. Schweizer F. Vallat A. Reymond P. García-Casado G. (2019). Jasmonate-related MYC Transcription Factors Are Functionally Conserved in *Marchantia polymorpha* . Plant Cell. 31, 2491–2509. 10.1105/tpc.18.00974 31391256 PMC6790078

[B98] Pham V. N. Kathare P. K. Huq E. (2018). Phytochromes and Phytochrome Interacting Factors. Plant Physiol. 176 (2), 1025–1038. 10.1104/pp.17.01384 29138351 PMC5813575

[B55] Pillitteri L. J. Sloan D. B. Bogenschutz N. L. Torii K. U. (2007). Termination of Asymmetric Cell Division and Differentiation of Stomata. Nature 445, 501–505. 10.3410/f.1056799.50874210.1038/nature05467 17183267

[B56] Rampey R. A. Woodward A. W. Hobbs B. N. Tierney M. P. Lahner B. Salt D. E. (2006). An Arabidopsis Basic Helix-Loop-Helix Leucine Zipper Protein Modulates Metal Homeostasis and Auxin Conjugate Responsiveness. Genetics 174, 1841–1857. 10.1534/genetics.106.061044 17028341 PMC1698629

[B97] Riechmann J. L. Heard J. Martin G. Reuber L. Jiang C. Z. Keddie J. (2000). Arabidopsis Transcription Factors: Genome-Wide Comparative Analysis among Eukaryotes. Science 290 (5499), 2105–2110. 10.1126/science.290.5499.2105 11118137

[B57] Roig-Villanova I. Bou-Torrent J. Galstyan A. Carretero-Paulet L. Portolés S. Rodríguez-Concepción M. (2007). Interaction of Shade Avoidance and Auxin Responses: a Role for Two Novel Atypical bHLH Proteins. EMBO J. 26, 4756–4767. 10.1038/sj.emboj.7601890 17948056 PMC2080812

[B58] Selote D. Samira R. Matthiadis A. Gillikin J. W. Long T. A. (2014). Iron-Binding E3 Ligase Mediates Iron Response in Plants by Targeting Basic Helix-Loop-Helix Transcription Factors. Plant Physiol. 167, 273–286. 10.1104/pp.114.250837 25452667 PMC4281009

[B59] Seo J. S. Joo J. Kim M. J. Kim Y. K. Nahm B. H. Song S. I. (2011). OsbHLH148, a Basic Helix-Loop-Helix Protein, Interacts with OsJAZ Proteins in a Jasmonate Signaling Pathway Leading to Drought Tolerance in Rice. Plant J. Cell. Mol. Biol. 65, 907–921. 10.1111/j.1365-313X.2010.04477.x 21332845

[B60] Sharpe R. M. Offermann S. (2014). One Decade after the Discovery of Single-Cell C_4_ Species in Terrestrial Plants: what Did We Learn about the Minimal Requirements of C_4_ Photosynthesis? Photosynth Res. 119, 169–180. 10.1007/s11120-013-9810-9 23494362

[B61] Shi R. Chiang V. L. (2005). Facile Means for Quantifying microRNA Expression by Real-Time PCR. Biotechniques 39, 519–525. 10.2144/000112010 16235564

[B62] Smith M. E. Koteyeva N. K. Voznesenskaya E. V. Okita T. W. Edwards G. E. (2009). Photosynthetic Features of Non-kranz Type C_4_ versus Kranz Type C_4_ and C_3_ Species in Subfamily Suaedoideae (Chenopodiaceae). Funct. Plant Biol. 36, 770–782. 10.1071/FP09120 32688687

[B63] Sorensen A.-M. Kröber S. Unte U. S. Huijser P. Dekker K. Saedler H. (2003). The Arabidopsis *ABORTED MICROSPORES (AMS)* Gene Encodes a MYC Class Transcription Factor. Plant J. 33, 413–423. 10.1046/j.1365-313X.2003.01644.x 12535353

[B64] Sun H. Fan H. J. Ling H. Q. (2015). Genome-wide Identification and Characterization of the *bHLH* Gene Family in Tomato. BMC genomics 16, 1–12. 10.1186/s12864-014-1209-2 25612924 PMC4312455

[B65] Szécsi J. Joly C. Bordji K. Varaud E. Cock J. M. Dumas C. (2006). BIGPETALp, a bHLH Transcription Factor Is Involved in the Control of *Arabidopsis* Petal Size. EMBO J. 25, 3912–3920. 10.1038/sj.emboj.7601270 16902407 PMC1553195

[B66] Thomashow M. F. (2001). So What's New in the Field of Plant Cold Acclimation? Lots!. Plant Physiol. 125, 89–93. 10.11042Fpp.125.1.8910.1104/pp.125.1.89 11154304 PMC1539333

[B67] Tian H. Guo H. Dai X. Cheng Y. Zheng K. Wang X. (2015). An ABA Down-Regulated *bHLH* Transcription Repressor Gene, *bHLH129* Regulates Root Elongation and ABA Response when Overexpressed in *Arabidopsis* . Sci. Rep. 5, 1–11. 10.1038/srep17587 PMC4667245 26625868

[B68] Tian M. Zhang X. Zhu Y. Xie G. Qin M. (2018). Global Transcriptome Analyses Reveal Differentially Expressed Genes of Six Organs and Putative Genes Involved in (Iso) flavonoid Biosynthesis in *Belamcanda chinensis* . Front. Plant Sci. 9, 1160. 10.3389/fpls.2018.01160 30154811 PMC6102373

[B69] Toledo-Ortiz G. Huq E. Quail P. H. (2003). The *Arabidopsis* Basic/Helix-Loop-Helix Transcription Factor Family. Plant Cell. 15, 1749–1770. 10.1105/tpc.013839 12897250 PMC167167

[B70] Trapnell C. Roberts A. Goff L. Pertea G. Kim D. Kelley D. R. (2012). Differential Gene and Transcript Expression Analysis of RNA-Seq Experiments with TopHat and Cufflinks. Nat. Protoc. 7, 562–578. 10.1038/nprot.2012.016 22383036 PMC3334321

[B71] Voznesenskaya E. V. Franceschi V. R. Edwards G. E. (2004). Light-dependent Development of Single Cell C_4_ Photosynthesis in Cotyledons of *Borszczowia aralocaspica* (Chenopodiaceae) during Transformation from a Storage to a Photosynthetic Organ. Ann. Bot. 93, 177–187. 10.1093/aob/mch026 14707001 PMC4241080

[B72] Voznesenskaya E. V. Franceschi V. R. Kiirats O. Freitag H. Edwards G. E. (2001). Kranz Anatomy Is Not Essential for Terrestrial C_4_ Plant Photosynthesis. Nature 414, 543–546. 10.1038/35107073 11734854

[B99] Walther D. Brunnemann R. Selbig J. (2007). The regulatory code for transcriptional response diversity and its relation to genome structural properties in *A. thaliana* . PLoS Genet. 3 (2), e11. 10.1371/journal.pgen.0030011 17291162 PMC1796623

[B73] Wang J. Cheng G. Wang C. He Z. Lan X. Zhang S. (2017). The bHLH Transcription Factor CgbHLH001 Is a Potential Interaction Partner of CDPK in Halophyte *Chenopodium glaucum* . Sci. Rep. 7, 1–16. 10.1038/s41598-017-06706-x 28814803 PMC5559460

[B74] Wang L. Huang Z. Baskin C. C. Baskin J. M. Dong M. (2008). Germination of Dimorphic Seeds of the Desert Annual Halophyte *Suaeda aralocaspica* (Chenopodiaceae), a C_4_ Plant without Kranz Anatomy. Ann. Bot. 102, 757–769. 10.1093/aob/mcn158 18772148 PMC2712381

[B75] Wang L. Ma G. Wang H. Cheng C. Mu S. Quan W. (2019a). A Draft Genome Assembly of Halophyte *Suaeda aralocaspica*, a Plant that Performs C_4_ Photosynthesis within Individual Cells. Gigascience 8, giz116. 10.1093/gigascience/giz116 31513708 PMC6741815

[B76] Wang L. Wang H. L. Yin L. Tian C. Y. (2017). Transcriptome Assembly in *Suaeda aralocaspica* to Reveal the Distinct Temporal Gene/miRNA Alterations between the Dimorphic Seeds during Germination. BMC Genomics 18, 1–21. 10.1186/s12864-017-4209-1 29052505 PMC5649071

[B77] Wang L. Xiang L. Hong J. Xie Z. Li B. (2019b). Genome-wide Analysis of bHLH Transcription Factor Family Reveals Their Involvement in Biotic and Abiotic Stress Responses in Wheat (*Triticum aestivum* L.). 3 Biotech. 9, 1–16. 10.1007/s13205-019-1742-4 PMC6536565 31139551

[B78] Wang P. Su L. Gao H. Jiang X. Wu X. Li Y. (2018). Genome-wide Characterization of *bHLH* Genes in Grape and Analysis of Their Potential Relevance to Abiotic Stress Tolerance and Secondary Metabolite Biosynthesis. Front. Plant Sci. 9, 64. 10.3389/fpls.2018.00064 29449854 PMC5799661

[B79] Wei K. Chen H. (2018). Comparative Functional Genomics Analysis of *bHLH* Gene Family in Rice, Maize and Wheat. BMC Plant Biol. 18, 1–21. 10.1186/s12870-018-1529-5 30497403 PMC6267037

[B80] Weissgerber T. L. Milic N. M. Winham S. J. Garovic V. D. (2015). Beyond Bar and Line Graphs: Time for a New Data Presentation Paradigm. PLoS Biol. 13, e1002128. 10.1371/journal.pbio.1002128 25901488 PMC4406565

[B81] Xing Q. Creff A. Waters A. Tanaka H. Goodrich J. Ingram G. C. (2013). *ZHOUPI* Controls Embryonic Cuticle Formation via a Signalling Pathway Involving the Subtilisin Protease ABNORMAL LEAF-SHAPE1 and the Receptor Kinases GASSHO1 and GASSHO2. Development 140, 770–779. 10.1242/dev.088898 23318634

[B82] Xu H. Wang N. Liu J. Qu C. Wang Y. Jiang S. (2017). The Molecular Mechanism Underlying Anthocyanin Metabolism in Apple Using the *MdMYB16* and *MdbHLH33* Genes. Plant Mol. Biol. 94, 149–165. 10.1007/s11103-017-0601-0 28286910

[B83] Xu J. Ding Z. Vizcay-Barrena G. Shi J. Liang W. Yuan Z. (2014). *ABORTED MICROSPORES* Acts as a Master Regulator of Pollen Wall Formation in *Arabidopsis* . Plant Cell. 26, 1544–1556. 10.1105/tpc.114.122986 24781116 PMC4036570

[B84] Xu Y. C. Niu X. M. Li X. X. He W. Chen J. F. Zou Y. P. (2019). Adaptation and Phenotypic Diversification in *Arabidopsis* through Loss-Of-Function Mutations in Protein-Coding Genes. Plant Cell. 31, 1012–1025. 10.1105/tpc.18.00791 30886128 PMC6533021

[B85] Yang S. Johnston N. Talideh E. Mitchell S. Jeffree C. Goodrich J. (2008). The Endosperm-specific *ZHOUPI* Gene of *Arabidopsis thaliana* Regulates Endosperm Breakdown and Embryonic Epidermal Development. Development 135, 3501–3509. 10.1242/dev.026708 18849529

[B86] Yin Y. Vafeados D. Tao Y. Yoshida S. Asami T. Chory J. (2005). A New Class of Transcription Factors Mediates Brassinosteroid-Regulated Gene Expression in *Arabidopsis* . Cell. 120, 249–259. 10.1016/j.cell.2004.11.044 15680330

[B87] Yue X. Zhang G. Zhao Z. Yue J. Pu X. Sui M. (2019). A Cryophyte Transcription Factor, CbABF1, Confers Freezing, and Drought Tolerance in Tobacco. Front. Plant Sci. 10, 699. 10.3389/fpls.2019.00699 31214219 PMC6555190

[B88] Zarka D. G. Vogel J. T. Cook D. Thomashow M. F. (2003). Cold Induction of *Arabidopsis CBF* Genes Involves Multiple ICE (Inducer of CBF Expression) Promoter Elements and a Cold-Regulatory Circuit that Is Desensitized by Low Temperature. Plant Physiol. 133, 910–918. 10.11042Fpp.103.02716910.1104/pp.103.027169 14500791 PMC219064

[B89] Zhang C. Feng R. Ma R. Shen Z. Cai Z. Song Z. (2018). Genome-wide Analysis of Basic Helix-Loop-Helix Superfamily Members in Peach. PLoS One 13, e0195974. 10.1371/journal.pone.0195974 29659634 PMC5901983

[B90] Zhang X. Y. Qiu J. Y. Hui Q. L. Xu Y. Y. He Y. Z. Peng L. Z. (2020). Systematic Analysis of the Basic/helix-Loop-Helix (bHLH) Transcription Factor Family in Pummelo (*Citrus grandis*) and Identification of the Key Members Involved in the Response to Iron Deficiency. BMC Genomics 21, 1–16. 10.1186/s12864-020-6644-7 PMC7071715 32171259

[B91] Zhang X. Dong J. Liu H. Wang J. Qi Y. Liang Z. (2016). Transcriptome Sequencing in Response to Salicylic Acid in *Salvia miltiorrhiza* . Plos One 11, e0147849. 10.1007/s11240-021-02135-x10.1371/journal.pone.0147849 26808150 PMC4726470

[B92] Zhang X. Luo H. Xu Z. Zhu Y. Ji A. Song J. (2015). Genome-wide Characterisation and Analysis of bHLH Transcription Factors Related to Tanshinone Biosynthesis in *Salvia miltiorrhiza* . Sci. Rep. 5, 1–10. 10.1038/srep11244 PMC4502395 26174967

[B93] Zhao P. Li X. Jia J. Yuan G. Chen S. Qi D. (2019). *bHLH92* from Sheepgrass Acts as a Negative Regulator of Anthocyanin/proanthocyandin Accumulation and Influences Seed Dormancy. J. Exp. Bot. 70, 269–284. 10.1093/jxb/erzo1710.1093/jxb/ery335 30239820 PMC6354636

[B94] Zheng K. Wang Y. Wang S. (2019). The Non-DNA Binding bHLH Transcription Factor Paclobutrazol Resistances Are Involved in the Regulation of ABA and Salt Responses in *Arabidopsis* . Plant Physiology Biochem. 139, 239–245. 10.1016/j.plaphy.2019.03.026 30921735

[B95] Zheng W. Guo J. Lu X. Qiao Y. Liu D. Pan S. (2022). cAMP-response Element Binding Protein Mediates Podocyte Injury in Diabetic Nephropathy by Targeting lncRNA DLX6-AS1. Metabolism 129, 155155. 10.1016/j.metabol.2022.155155 35093327

[B96] Zhou Z. Wang J. Zhang S. Yu Q. Lan H. (2020). Investigation of the Nature of CgCDPK and CgbHLH001 Interaction and the Function of bHLH Transcription Factor in Stress Tolerance in *Chenopodium glaucum* . Front. Plant Sci. 11, 2173. 10.3389/fpls.2020.603298 PMC7862342 33552098

