# Supplementary material for: Genome-Wide Characterization and Analysis of the bHLH Transcription Factor Family in Suaeda aralocaspica, an Annual Halophyte With Single-Cell C4 Anatomy
Source: Front Genet. 2022 Jul 7;13:927830. doi: 10.3389/fgene.2022.927830 (PMC9301494; doi:10.3389/fgene.2022.927830)
Supplement: Supplementary file 1 [file Table1.docx]

**Table 1** Characteristics of *bHLH* gene family in *S. aralocaspica*.

| Gene name | Genome ID | GeneBank | ORF (bp) | *PI* | MW (kDa) | Size (aa) | Subcellular location |
| --- | --- | --- | --- | --- | --- | --- | --- |
| SabHLH001 | GOSA_00000076-RA | ON400862 | 1,668 | 5.74 | 60.27532 | 556 | Nucleus |
| SabHLH002 | GOSA_00000223-RA | ON400863 | 1,383 | 6.67 | 50.40351 | 461 | Nucleus |
| SabHLH003 | GOSA_00000435-RA | ON400865 | 1,431 | 4.72 | 53.86281 | 477 | Nucleus |
| SabHLH004 | GOSA_00000601-RA | ON400861 | 2,037 | 6.16 | 74.15591 | 679 | Nucleus |
| SabHLH005 | GOSA_00000643-RA | ON400860 | 1,431 | 6.28 | 52.52881 | 477 | Nucleus |
| SabHLH006 | GOSA_00000671-RA | ON400866 | 1,020 | 8.66 | 38.82797 | 340 | Nucleus |
| SabHLH007 | GOSA_00000723-RA | ON400867 | 885 | 6.13 | 31.33137 | 295 | Nucleus |
| SabHLH008 | GOSA_00001077-RA | ON400864 | 1,122 | 5.71 | 41.83251 | 374 | Nucleus |
| SabHLH009 | GOSA_00001306-RA | ON400858 | 891 | 7.75 | 33.91263 | 297 | Nucleus |
| SabHLH010 | GOSA_00001307-RA | ON400856 | 852 | 5.55 | 31.92971 | 284 | Nucleus |
| SabHLH011 | GOSA_00001444-RA | ON400857 | 648 | 6.36 | 24.71007 | 216 | Nucleus |
| SabHLH012 | GOSA_00001689-RA | ON400859 | 1,992 | 5.91 | 72.36971 | 664 | Nucleus |
| SabHLH013 | GOSA_00001701-RA | ON400855 | 906 | 8.6 | 34.04228 | 302 | Nucleus |
| SabHLH014 | GOSA_00001967-RA | ON400888 | 1,494 | 5.24 | 53.81603 | 498 | Nucleus |
| SabHLH015 | GOSA_00001974-RA | ON400884 | 576 | 6.85 | 21.2707 | 192 | Nucleus |
| SabHLH016 | GOSA_00002069-RA | ON400887 | 1,188 | 5.98 | 45.26038 | 396 | Nucleus |
| SabHLH017 | GOSA_00002373-RA | ON400883 | 1,125 | 5.62 | 41.93782 | 375 | Nucleus |
| SabHLH018 | GOSA_00002416-RA | ON400885 | 1,362 | 7.1 | 51.12012 | 454 | Nucleus |
| SabHLH019 | GOSA_00002694-RA | ON400886 | 1,341 | 6.19 | 47.16991 | 447 | Nucleus |
| SabHLH020 | GOSA_00002849-RA | ON400893 | 735 | 5.39 | 27.55012 | 245 | Nucleus |
| SabHLH021 | GOSA_00003472-RA | ON400892 | 624 | 9.17 | 23.1888 | 208 | Nucleus |
| SabHLH022 | GOSA_00003586-RA | ON400872 | 843 | 8.29 | 31.46418 | 281 | Nucleus |
| SabHLH023 | GOSA_00003869-RA | ON400871 | 1,053 | 8.34 | 38.86735 | 351 | Nucleus |
| SabHLH024 | GOSA_00004355-RA | ON400904 | 273 | 7.94 | 10.50083 | 91 | Nucleus |
| SabHLH025 | GOSA_00004508-RA | ON400876 | 1,005 | 4.9 | 37.11856 | 335 | Nucleus |
| SabHLH026 | GOSA_00004680-RA | ON400877 | 1,515 | 7.09 | 55.49633 | 505 | Nucleus |
| SabHLH027 | GOSA_00004781-RA | ON400878 | 1,983 | 4.64 | 75.61593 | 661 | Nucleus |
| SabHLH028 | GOSA_00004882-RA | ON400879 | 1,263 | 8.93 | 44.33566 | 421 | Nucleus |
| SabHLH029 | GOSA_00005410-RA | ON400896 | 1,206 | 6.05 | 44.62922 | 402 | Nucleus |
| SabHLH030 | GOSA_00005475-RA | ON400897 | 945 | 8.79 | 35.53922 | 315 | Nucleus |
| SabHLH031 | GOSA_00006132-RA | ON400869 | 927 | 6.16 | 33.86087 | 309 | Nucleus |
| SabHLH032 | GOSA_00006633-RA | ON400870 | 777 | 5.49 | 28.83567 | 259 | Nucleus |
| SabHLH033 | GOSA_00006634-RA | ON400868 | 822 | 5.26 | 30.63455 | 274 | Nucleus |
| SabHLH034 | GOSA_00006830-RA | ON400913 | 1,257 | 5.76 | 46.51908 | 419 | Nucleus |
| SabHLH035 | GOSA_00007033-RA | ON400912 | 726 | 7.58 | 26.98551 | 242 | Nucleus |
| SabHLH036 | GOSA_00007093-RA | ON400850 | 1,131 | 6.27 | 41.48272 | 377 | Nucleus |
| SabHLH037 | GOSA_00007353-RA | ON400851 | 2,067 | 6.45 | 74.6124 | 689 | Nucleus |
| SabHLH038 | GOSA_00008204-RA | ON400849 | 927 | 6.4 | 32.26992 | 309 | Chloroplast\Nucleus |
| SabHLH039 | GOSA_00008334-RA | ON400848 | 1,254 | 5.14 | 47.27305 | 418 | Nucleus |
| SabHLH040 | GOSA_00008687-RA | ON400874 | 963 | 5.96 | 35.63039 | 321 | Nucleus |
| SabHLH041 | GOSA_00008720-RA | ON400875 | 1,011 | 6.26 | 36.6356 | 337 | Nucleus |
| SabHLH042 | GOSA_00010211-RA | ON400903 | 1,206 | 6.1 | 44.07468 | 402 | Nucleus |
| SabHLH043 | GOSA_00010462-RA | ON400902 | 780 | 9.38 | 28.2595 | 260 | Nucleus |
| SabHLH044 | GOSA_00010702-RA | ON400889 | 1,980 | 5.31 | 72.66926 | 660 | Cytoplasm |
| SabHLH045 | GOSA_00010732-RA | ON400891 | 1,206 | 6.54 | 45.62066 | 402 | Nucleus |
| SabHLH046 | GOSA_00010765-RA | ON400890 | 987 | 4.76 | 37.56619 | 329 | Nucleus |
| SabHLH047 | GOSA_00011122-RA | ON400919 | 1,209 | 5.56 | 42.58493 | 403 | Nucleus |
| SabHLH048 | GOSA_00011171-RA | ON400918 | 258 | 4.47 | 10.03095 | 86 | Nucleus |
| SabHLH049 | GOSA_00011177-RA | ON400916 | 762 | 5.4 | 29.04995 | 254 | Nucleus |
| SabHLH050 | GOSA_00011282-RA | ON400917 | 690 | 6.6 | 25.60963 | 230 | Nucleus |
| SabHLH051 | GOSA_00011283-RA | ON400920 | 960 | 6.13 | 35.77709 | 320 | Nucleus |
| SabHLH052 | GOSA_00011847-RA | ON400911 | 1,194 | 8.63 | 43.00472 | 389 | Nucleus |
| SabHLH053 | GOSA_00011938-RA | ON400910 | 1,389 | 9.43 | 50.93376 | 463 | Nucleus |
| SabHLH054 | GOSA_00012457-RA | ON400926 | 960 | 5.36 | 35.7185 | 320 | Nucleus |
| SabHLH055 | GOSA_00012983-RA | ON400923 | 792 | 5.48 | 30.25622 | 264 | Nucleus |
| SabHLH056 | GOSA_00013009-RA | ON400922 | 390 | 5.06 | 14.57895 | 130 | Nucleus |
| SabHLH057 | GOSA_00013011-RA | ON400921 | 804 | 8.37 | 30.67592 | 268 | Nucleus |
| SabHLH058 | GOSA_00013158-RA | ON400908 | 1,017 | 5.35 | 37.85607 | 339 | Nucleus |
| SabHLH059 | GOSA_00013161-RA | ON400907 | 963 | 4.95 | 36.1451 | 321 | Nucleus |
| SabHLH060 | GOSA_00013229-RA | ON400906 | 1,203 | 6.24 | 43.18755 | 401 | Nucleus |
| SabHLH061 | GOSA_00013409-RA | ON400894 | 471 | 10.91 | 17.91746 | 157 | Nucleus |
| SabHLH062 | GOSA_00014741-RA | ON400873 | 1,584 | 9.3 | 59.38059 | 528 | Nucleus |
| SabHLH063 | GOSA_00015033-RA | ON400852 | 933 | 5.93 | 33.2459 | 311 | Nucleus |
| SabHLH064 | GOSA_00015228-RA | ON400914 | 1,479 | 5.57 | 55.14247 | 493 | Nucleus |
| SabHLH065 | GOSA_00015812-RA | ON400905 | 441 | 10.26 | 16.50293 | 147 | Nucleus |
| SabHLH066 | GOSA_00016476-RA | ON400909 | 813 | 7.63 | 31.04324 | 271 | Nucleus |
| SabHLH067 | GOSA_00018427-RA | ON400853 | 1,008 | 5.68 | 37.65921 | 336 | Nucleus |
| SabHLH068 | GOSA_00018432-RA | ON400854 | 1,068 | 6.95 | 39.4324 | 356 | Nucleus |
| SabHLH069 | GOSA_00018520-RA | ON400887 | 828 | 9.14 | 30.15356 | 276 | Nucleus |
| SabHLH070 | GOSA_00018672-RA | ON400927 | 1,023 | 6.27 | 37.64574 | 341 | Nucleus |
| SabHLH071 | GOSA_00018673-RA | ON400928 | 1,188 | 5.84 | 33.1677 | 296 | Nucleus |
| SabHLH072 | GOSA_00018924-RA | ON400929 | 1,017 | 5.73 | 37.30524 | 339 | Nucleus |
| SabHLH073 | GOSA_00019477-RA | ON400901 | 765 | 5.77 | 27.0998 | 255 | Nucleus |
| SabHLH074 | GOSA_00019526-RA | ON400898 | 1,503 | 5.97 | 55.38031 | 501 | Nucleus |
| SabHLH075 | GOSA_00019527-RA | ON400900 | 1,530 | 5.97 | 56.18658 | 510 | Nucleus |
| SabHLH076 | GOSA_00019547-RA | ON400899 | 2,097 | 5.96 | 77.3953 | 699 | Nucleus |
| SabHLH077 | GOSA_00020011-RA | ON400924 | 1,071 | 4.83 | 38.69557 | 357 | Nucleus |
| SabHLH078 | GOSA_00020455-RA | ON400895 | 1,929 | 5.43 | 73.52925 | 643 | Nucleus |
| SabHLH079 | GOSA_00020680-RA | ON400925 | 1,791 | 5.3 | 65.85017 | 597 | Nucleus |
| SabHLH080 | GOSA_00022456-RA | ON400882 | 1,416 | 5.47 | 52.09624 | 472 | Nucleus |
| SabHLH081 | GOSA_00022501-RA | ON400880 | 1,593 | 5.62 | 57.19449 | 531 | Nucleus |
| SabHLH082 | GOSA_00022531-RA | ON400881 | 747 | 6.61 | 28.33789 | 249 | Nucleus |
| SabHLH083 | GOSA_00026973-RA | ON400915 | 945 | 7 | 35.06321 | 315 | Nucleus |

**Supplementary Table 1**. *bHLH* gene information of *Arabidopsis* used to construct the phylogenetic tree.

| **Species** | **Gene ID** |  | **Gene ID** |  |
| --- | --- | --- | --- | --- |
| *Arabidopsis thaliana* | AT5G41315 | AtbHLH001 | AT5G58010 | AtbHLH082 |
|  | AT1G63650 | AtbHLH002 | AT1G66470 | AtbHLH083 |
|  | AT4G16430 | AtbHLH003 | AT2G14760 | AtbHLH084 |
|  | AT4G17880 | AtbHLH004 | AT4G33880 | AtbHLH085 |
|  | AT5G46760 | AtbHLH005 | AT5G37800 | AtbHLH086 |
|  | AT1G32640 | AtbHLH006 | AT3G21330 | AtbHLH087 |
|  | AT1G03040 | AtbHLH007 | AT5G67060 | AtbHLH088 |
|  | AT1G09530 | AtbHLH008 | AT1G06170 | AtbHLH089 |
|  | AT2G43010 | AtbHLH009 | AT1G10610 | AtbHLH090 |
|  | AT2G31220 | AtbHLH010 | AT2G31210 | AtbHLH091 |
|  | AT4G36060 | AtbHLH011 | AT5G43650 | AtbHLH092 |
|  | AT4G00480 | AtbHLH012 | AT5G65640 | AtbHLH093 |
|  | AT1G01260 | AtbHLH013 | AT1G22490 | AtbHLH094 |
|  | AT4G00870 | AtbHLH014 | AT1G49770 | AtbHLH095 |
|  | AT2G20180 | AtbHLH015 | AT1G72210 | AtbHLH096 |
|  | AT4G00050 | AtbHLH016 | AT3G24140 | AtbHLH097 |
|  | AT2G46510 | AtbHLH017 | AT5G53210 | AtbHLH098 |
|  | AT2G22750 | AtbHLH018 | AT5G65320 | AtbHLH099 |
|  | AT2G22760 | AtbHLH019 | AT2G41240 | AtbHLH100 |
|  | AT2G22770 | AtbHLH020 | AT5G04150 | AtbHLH101 |
|  | AT2G16910 | AtbHLH021 | AT1G69010 | AtbHLH102 |
|  | AT4G21330 | AtbHLH022 | AT4G21340 | AtbHLH103 |
|  | AT4G28790 | AtbHLH023 | AT4G14410 | AtbHLH104 |
|  | AT4G36930 | AtbHLH024 | AT5G54680 | AtbHLH105 |
|  | AT4G37850 | AtbHLH025 | AT2G41130 | AtbHLH106 |
|  | AT1G02340 | AtbHLH026 | AT3G56770 | AtbHLH107 |
|  | AT4G29930 | AtbHLH027 | AT1G25310 | AtbHLH108 |
|  | AT5G46830 | AtbHLH028 | AT1G68240 | AtbHLH109 |
|  | AT2G28160 | AtbHLH029 | AT1G27660 | AtbHLH110 |
|  | AT1G68810 | AtbHLH030 | AT1G31050 | AtbHLH111 |
|  | AT1G59640 | AtbHLH031 | AT1G61660 | AtbHLH112 |
|  | AT3G25710 | AtbHLH032 | AT3G19500 | AtbHLH113 |
|  | AT1G12860 | AtbHLH033 | AT4G05170 | AtbHLH114 |
|  | AT3G23210 | AtbHLH034 | AT1G51070 | AtbHLH115 |
|  | AT5G57150 | AtbHLH035 | AT3G26744 | AtbHLH116 |
|  | AT5G51780 | AtbHLH036 | AT3G22100 | AtbHLH117 |
|  | AT3G50330 | AtbHLH037 | AT4G25400 | AtbHLH118 |
|  | AT3G56970 | AtbHLH038 | AT4G28811 | AtbHLH119 |
|  | AT3G56980 | AtbHLH039 | AT5G51790 | AtbHLH120 |
|  | AT4G00120 | AtbHLH040 | AT3G19860 | AtbHLH121 |
|  | AT5G56960 | AtbHLH041 | AT1G51140 | AtbHLH122 |
|  | AT4G09820 | AtbHLH042 | AT3G20640 | AtbHLH123 |
|  | AT5G09750 | AtbHLH043 | AT2G46970 | AtbHLH124 |
|  | AT1G18400 | AtbHLH044 | AT1G62975 | AtbHLH125 |
|  | AT3G06120 | AtbHLH045 | AT4G25410 | AtbHLH126 |
|  | AT5G08130 | AtbHLH046 | AT4G28815 | AtbHLH127 |
|  | AT3G47640 | AtbHLH047 | AT1G05805 | AtbHLH128 |
|  | AT2G42300 | AtbHLH048 | AT2G43140 | AtbHLH129 |
|  | AT1G68920 | AtbHLH049 | AT2G42280 | AtbHLH130 |
|  | AT1G73830 | AtbHLH050 | AT4G38071 | AtbHLH131 |
|  | AT2G40200 | AtbHLH051 | AT3G62090 | AtbHLH132 |
|  | AT1G30670 | AtbHLH052 | AT2G20095 | AtbHLH133 |
|  | AT2G34820 | AtbHLH053 | AT5G15160 | AtbHLH134 |
|  | AT1G27740 | AtbHLH054 | AT1G74500 | AtbHLH135 |
|  | AT1G12540 | AtbHLH055 | AT5G39860 | AtbHLH136 |
|  | AT4G28800 | AtbHLH056 | AT5G50915 | AtbHLH137 |
|  | AT4G01460 | AtbHLH057 | AT2G31215 | AtbHLH138 |
|  | AT4G36540 | AtbHLH058 | AT5G43175 | AtbHLH139 |
|  | AT4G02590 | AtbHLH059 | AT5G01310 | AtbHLH140 |
|  | AT3G57800 | AtbHLH060 | AT5G38860 | AtbHLH141 |
|  | AT5G10570 | AtbHLH061 | AT5G64340 | AtbHLH142 |
|  | AT3G07340 | AtbHLH062 | AT5G09460 | AtbHLH143 |
|  | AT4G34530 | AtbHLH063 | AT1G29950 | AtbHLH144 |
|  | AT2G18300 | AtbHLH064 | AT5G50010 | AtbHLH145 |
|  | AT3G59060 | AtbHLH065 | AT4G30180 | AtbHLH146 |
|  | AT2G24260 | AtbHLH066 | AT3G17100 | AtbHLH147 |
|  | AT3G61950 | AtbHLH067 | AT3G06590 | AtbHLH148 |
|  | AT4G29100 | AtbHLH068 | AT1G09250 | AtbHLH149 |
|  | AT4G30980 | AtbHLH069 | AT3G05800 | AtbHLH150 |
|  | AT2G46810 | AtbHLH070 | AT2G47270 | AtbHLH151 |
|  | AT5G46690 | AtbHLH071 | AT1G22380 | AtbHLH152 |
|  | AT5G61270 | AtbHLH072 | AT1G05710 | AtbHLH153 |
|  | AT5G67110 | AtbHLH073 | AT2G31730 | AtbHLH154 |
|  | AT1G10120 | AtbHLH074 | AT2G31280 | AtbHLH155 |
|  | AT1G25330 | AtbHLH075 | AT2G27230 | AtbHLH156 |
|  | AT1G26260 | AtbHLH076 | AT1G64625 | AtbHLH157 |
|  | AT3G23690 | AtbHLH077 | AT2G43060 | AtbHLH158 |
|  | AT5G48560 | AtbHLH078 | AT4G30410 | AtbHLH159 |
|  | AT5G62610 | AtbHLH079 | AT1G71200 | AtbHLH160 |
|  | AT1G35460 | AtbHLH080 | AT3G47710 | AtbHLH161 |
|  | AT4G09180 | AtbHLH081 | AT4G20970 | AtbHLH162 |

**Supplementary Table 2**. Primer sequences used in the present study.

| **Primer name** | **Primer sequence（5'-3'）** |
| --- | --- |
| *SabHLH011*-qRT-F | CTAGGCTTTCGACCGACCC |
| *SabHLH011*-qRT-R | ACCACCAGGGATTAGGCTTTG |
| *SabHLH014*-qRT-F | TGCTTAGGTCGGTTGTGCCT |
| *SabHLH014*-qRT-R | CGGCATGGAATAGTGGGTGGG |
| *SabHLH026*-qRT-F | TGCCAGGGTGGATCGGAAAAC |
| *SabHLH026*-qRT-R | GCGATCTCTTCGTCGCCTCT |
| *SabHLH028*-qRT-F | AGGGCTTCCATACTGGGGGA |
| *SabHLH028*-qRT-R | ATTCGAGGGGGCAAGTTCGG |
| *SabHLH037*-qRT-F | TCAGGGAATGCCGGGGTCTAA |
| *SabHLH037*-qRT-R | TTCCTGGCATTGGAGCAGTGG |
| *SabHLH038*-qRT-F | GGTCAAGTGCTGATGGCTCGT |
| *SabHLH038*-qRT-R | TGGCAAGCCCAAGCCCATT |
| *SabHLH048*-qRT-F | GACGACGATTCGAGCTCCCC |
| *SabHLH048*-qRT-R | GAGCAAAGAGGCGTTCATTGAGT |
| *SabHLH059*-qRT-F | CCCTTGTTGCTTCTGTTTTCCCTG |
| *SabHLH059*-qRT-R | TCAGCTTGGACTTCTGGTGCAT |
| *SabHLH169(076)*-qRT-F | GCCTGCGAGCCATTTTGGTC |
| *SabHLH169(076*)-qRT-R | CAATTCCACGGCCTCGCTGA |
| *SaTUB*-qRT-F | CCTTATTCCATTCCCCAGGCTTC |
| *SaTUB*-qRT-R | CATCTGCTCATCAACCTCCTTTGTGC |
| *SabHLH169(076)*-superF | GCGTCGACATGGCTACTCACTTGCAGCAGTTGCTTC |
| *SabHLH169(076)*-superR-GFP | GGGGTACCGGCATTAGTTTTTGATTGAAGTAGTTGG |
| pGBKT7-*SabHLH169(076)*-F | TGCCATTGGCATGGCTACTCACTTGCAGCAGTTGCTTC |
| pGBKT7-*SabHLH169(076)*-R | CGGGATCCTCAGGCATTAGTTTTTGATTGAAGTAGTTGG |

**Supplementary Table 3**. Summary of the predicted functions of SabHLHs by comparative analysis with AtbHLHs

| **SabHLH** | **AtbHLH** | **Short name of function in Arabidopsis** | **Function in Arabidopsis** |
| --- | --- | --- | --- |
| SabHLH006 | AtbHLH057 | LHWL2 | LONESOME HIGHWAY-like protein 2 |
| SabHLH021 | AtbHLH045 | MUTE | Control sequential cell fate specification during stomatal differentiation (Pillitteri et al., 2007) |
| SabHLH030 | AtbHLH095 | RGE1 | Transcription factor that controls embryo growth  Regulates endosperm breakdown and embryonic epidermal development (Kondou et al., 2008; Yang et al., 2008; Xing et al., 2013) |
| SabHLH035 | AtbHLH105 | ILR3 | Modulate metal homeostasis and auxin-conjugate metabolism (Rampey et al., 2006) |
| SabHLH040 | AtbHLH102 | BIM2 | Implicated in brassinosteroid signaling (Yin et al., 2005) |
| SabHLH061 | AtbHLH043 | HEC3 | Redundantly control the development of the transmitting tract and stigma; each of these proteins can form heterodimers with SPATULA (Gremski et al., 2007) |
| SabHLH065 | AtbHLH029 | FIT | Essential protein involved in iron uptake responses (Colangelo and E., 2004)if |
| SabHLH073 | AtbHLH059 | UNE12 | Required for ovule fertilization (Pagnussat et al., 2005) |
| SabHLH076 | AtbHLH155 | LHWL3 | LONESOME HIGHWAY-like protein 3 |
| SabHLH078 | AtbHLH021 | AMS | Plays a crucial role in tapetum development (Sorensen et al., 2003) |
| SabHLH079 | AtbHLH097 | FAMA | Together with SPCH and MUTE, regulates the stomata formation (Ohashi-Ito and Bergmann, 2006) |
