# Supplementary figures and images for: Genome-Wide Characterization and Analysis of the bHLH Transcription Factor Family in Suaeda aralocaspica, an Annual Halophyte With Single-Cell C4 Anatomy
Source: Front Genet. 2022 Jul 7;13:927830. doi: 10.3389/fgene.2022.927830 (PMC9301494; doi:10.3389/fgene.2022.927830)

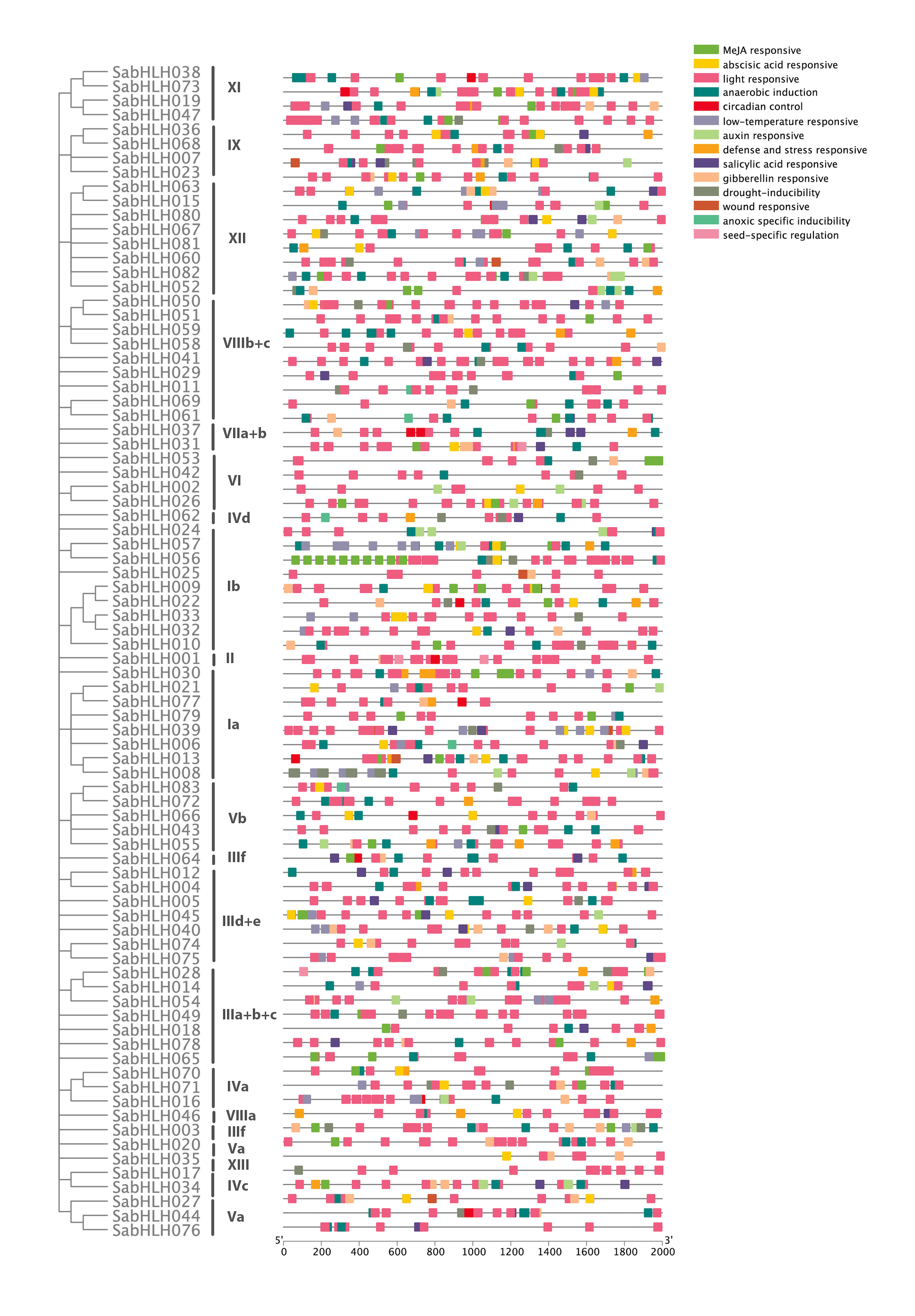

Supplement: Supplementary file 2 [file Image1.TIF]
